# Dual Nurr1/RXR agonism of valerenic acid and synthetic mimetics enables dimer-selective Nurr1 modulation

**DOI:** 10.1021/acsmedchemlett.5c00572

**Published:** 2025-12-09

**Authors:** Katharina Scholz, Úrsula López-García, Romy Busch, Julian A. Marschner, Daniel Merk

**Affiliations:** 1 Ludwig-Maximilians-Universität München, Department of Pharmacy, 81377 Munich, Germany

**Keywords:** Transcription factors, nuclear receptor related 1, retinoid X receptor, heterodimer, dual ligand, neurodegeneration

## Abstract

Nuclear receptor related 1 (Nurr1) is a neuroprotective transcription factor emerging as promising target in Parkinson’s disease and multiple sclerosis. It can act in three oligomeric forms as monomer, homodimer and heterodimer on different DNA response elements. We hypothesized that dual Nurr1 and RXR activation might enable selective modulation of Nurr1 in its heterodimeric form. A search for dual ligands revealed valerenic acid and a synthetic mimetic as Nurr1 and RXR activators. Biochemical and cellular characterization demonstrated that dual agonism destabilized the Nurr1 homodimer but left the heterodimer intact which translated into selective activation of the heterodimer response element in cells. In neuronal cells, a dual Nurr1/RXR agonist enhanced expression of only a subset of Nurr1 agonist induced genes providing initial proof-of-concept for the dimer-directed selective Nurr1 modulation approach.

The ligand-activated transcription factor nuclear receptor related 1 (Nurr1, NR4A2) is considered as a promising target in neurodegenerative diseases for its neuroprotective and anti-neuroinflammatory activities^1–4^. It is involved in (dopaminergic) neuron differentiation, maturation and survival^1,5^ by regulating genes of dopamine metabolism, neurotransmission, axon development and mitochondrial function^1,5–7^. In microglia, Nurr1 can be induced as an anti-inflammatory response and affects the inflammatory phenotype^8–12^. Moreover, both Alzheimer’s and Parkinson’s disease patients and animal models displayed diminished Nurr1 expression, and low Nurr1 levels were found, for example, to enhance susceptibility of neurons to α-synuclein induced damage^13–15^. These observations supporting therapeutic potential of Nurr1 activation have fueled ligand development and several Nurr1 modulators have recently been reported^16–19^.

Nurr1 can act in three different oligomeric forms – as monomer, as homodimer, and as heterodimer with the retinoid X receptor (RXR) as partner – which have been reported to signal via different DNA response elements (REs)^20–22^: The Nurr1 monomer interacts with the nerve growth factor-inducible-β-binding (NGFI-B) response element (NBRE), the homodimer binds to the Nur-responsive element (NurRE), and the heterodimer acts via DR5 or IR5 motifs^23^. Mechanistic studies on Nurr1-RXR interaction^23,24^ suggested that presence of RXR can inhibit Nurr1 signaling via the monomer response element NBRE and enhance activity on heterodimer REs. Moreover, Nurr1:RXR heterodimers were found to be activated by RXR agonists^21,25^ and selective RXR modulators displaying preference for the Nurr1:RXR dimer have been developed^26–29^ suggesting that Nurr1 activity is at least in part RXR regulated. On the other hand, recent studies indicated that ligand-induced release of the (transcriptionally most active) Nurr1 monomer from dimers contributes to the activation mechanism of Nurr1 agonists^30,31^. Taken together, these observations point to two avenues to chemical perturbation of Nurr1 activity via the receptor itself or its dimer partner RXR. Therefore, we hypothesized that dual agonism on both heterodimer partners Nurr1 and RXR could result in interference of these effects and potentially enable oligomer-selective control over Nurr1.

To identify dual Nurr1/RXR activators to probe this hypothesis and further decipher the interplay of both nuclear receptors, we tested our collection of Nurr1 and RXR activators for modulation of the respective other receptor. While the majority of tested compounds displayed strong selectivity for their original target, we discovered Nurr1 activation by valerenic acid (**1**; Table 1), which is also an RXR agonist^32^. The natural product thus emerged as valuable starting point to access dual agonists. However, Nurr1 activation efficacy by **1** was modest (1.3-fold max. activation) and required improvement for the potential use as a tool, prompting us to explore related compounds. While the natural hydroxy- and acetoxy-valerenic acid derivatives failed to activate Nurr1 and RXR^33^ (Figure S1), the synthetic mimetic **2**, obtained by computational de novo design with **1** as template^32^, displayed strong and balanced agonism on Nurr1 and RXR.

Encouraged by this appealing dual activity, we additionally evaluated close analogues of **2** containing modifications in the hydrophobic tetrahydroindole and carboxylic acid substructures (**3**-**5**). Compounds **2** and **4** were prepared (Scheme 1) in a one-pot multicomponent reaction as described previously for **2**^32^ using 2-(4-aminophenyl)acetic acid (**6**) or thereof prepared 2-(4-aminophenyl)acetamide (**7**)^34^, 4,4-difluorocyclohexanone (**8**) and acetoin (**9**). The aromatic compounds **3** and **5** were prepared by oxidative aromatization of their respective tetrahydroindoles **2** and **4** through prolonged stirring under air.

The computational design^32^ **2** mediated considerable 3.3-fold Nurr1 activation and exceeded valerenic acid in potency on both Nurr1 and RXR. Further improvement in dual Nurr1/RXR agonism was achieved by aromatization of the 5,5-difluorotetrahydroindole in **2** to the 5-fluoroindole **3**. RXR agonist potency of **3** increased by two- (RXRα/γ) to eight-fold (RXRβ) compared to **2** with no relevant change in efficacy. On Nurr1, **3** displayed particularly strong activation (> 4-fold) exceeding **2** and most available Nurr1 agonists^3,35^. Additionally, **3** provided improved chemical stability compared to **2**. Dual engagement of both targets by **2** and **3** was also evident in isothermal titration calorimetry (ITC) which revealed sub-micromolar affinity to the recombinant ligand binding domains (LBD) of both nuclear receptors. As RXR agonist binding typically involves a strong ionic interaction of a carboxylic acid motif in the ligand with an arginine residue, we evaluated the impact of replacing the carboxylate motifs of **2** and **3** by a primary amide (**4, 5**). The amide analogues indeed failed to activate RXR and, interestingly, also lost agonist activity on Gal4-Nurr1 suggesting a possible involvement of a neutralizing interaction with Nurr1, too. **2** and **3** thus emerged as best suitable early tools to explore the interplay of Nurr1 with RXR and the cellular impact of dual activation.

To determine the molecular response of the Nurr1:RXR heterodimer on dual agonist binding, we employed homogenous time-resolved fluorescence resonance energy transfer (HTRF) based assays with labeled Nurr1 and RXR LBD proteins. Tb^3+^-cryptate as streptavidin conjugate served as FRET donor and was coupled to biotinylated proteins. sGFP fusion proteins of the receptors of interest were used as FRET acceptors. Ligand effects on dimer formation were determined by titrating the sGFP-labeled dimer partner with a fixed concentration of the Tb^3+^-labeled counterpart. The agonists **2** and **3** markedly diminished Nurr1 homodimer formation (Figure 1a) in line with previous reports that suggested release of the transcriptionally more active monomer as activation mechanism by agonists^30,31^. The same effect was also observable for the natural product **1** (Figure S3). Diminished dimer strength was also evident for the Nurr1:RXR heterodimer in presence of a selective RXR agonist (SR11237) or a selective Nurr1 agonist (compound 1 from ^31^; Figure S4)^31^, while the dual agonists **2** and **3** left the dimer intact (Figure 1b). These results thus revealed a peculiar molecular impact of dual Nurr1/RXR agonists on the heterodimer that could possibly mediate selective activation.

Based on these findings, we next determined the effects of **2** and **3** on Nurr1 activity in a cellular setting using reporter constructs for the NGFI-B response element (NBRE), the Nur response element (NurRE), and the direct repeat 5 (DR5)^36^. All three response elements are robustly activated by common Nurr1 agonists^4,31^. The dual Nurr1/RXR agonists **2** and **3**, in contrast, only enhanced DR5 dependent transcription whereas no activation was detectable on NBRE and NurRE (Figure 1c). The RXR antagonist HX531^37^ counteracted the effects of **2** and **3** on DR5 (Figure 1c) supporting involvement of RXR in DR5 activation by the dual agonists.

As the molecular selectivity towards destabilizing the Nurr1 homodimer while sparing the Nurr1:RXR heterodimer thus translated into functional selectivity in cellular setting for activation of the heterodimer response element DR5, we next evaluated whether this selective Nurr1:RXR heterodimer activation resulted in distinct effects on gene expression compared to common Nurr1 agonists. Nurr1 is a key factor of neuroprotection and health of dopaminergic neurons. Therefore, we explored the impact of **2** (10 μM) and **3** (1 μM) on gene expression in rat dopaminergic neuronal cells (N27), which have been widely used to study Nurr1 agonists^17,18,38,39^. Both compounds were non-toxic at the test concentrations in a multiplex toxicity assay (Figure S5). The dual agonists robustly upregulated mRNA expression of only a subset of genes that were induced by a selective Nurr1 agonist (Figure 1d). While the expression levels of tyrosine hydroxylase (TH), fibronectin leucine rich transmembrane protein 2 (FLRT2), brain-derived neurotrophic factor (BDNF) and collapsin response mediator protein 4 (CRMP4; Dpysl3 in rat) displayed similar responses to the dual agonists and the reference Nurr1 agonist, X-linked inhibitor of apoptosis protein (XIAP), sestrin 3 (SESN3), cyclin D2 (CCND2), baculoviral inhibitor of apoptosis repeat-containing 5 (BIRC5), and superoxide dismutase 2 (SOD2) were not induced by the dual agonists.

These preliminary insights into the cellular impact of dual Nurr1/RXR agonism pointed to selective modulation of Nurr1 regulated gene expression. In a promoter analysis (-2000 to +200 bp from TSS) of the studied genes for Nurr1 REs using JASPAR^40^ (Fig. 1e), empirical probabilities for the presence of DR5 in the promoter regions of genes that were induced by **2** and **3** tended to be higher whereas NBRE was frequent in all analyzed promoters. However, BDNF and CCND2 were notable exceptions, and the promoter analysis has limitations as the detection of response sequences with JASPAR relies on probabilities for the arrangement of bases in REs. These annotated probability matrices may be incomplete, and the defined tolerance may be inappropriate. Additionally, our experiments were conducted in a rat cell line and Nurr1 REs for this species are not annotated in JASPAR, but comparison of Nurr1 REs from other available species suggested strong conservation. Nevertheless, the selective gene expression effects of **2** and **3** and the partially aligning predicted presence of DR5 REs in the promoter regions of the respective genes support the hypothesis that heterodimer selective Nurr1 modulation is feasible. A possible contribution of further mechanisms in the observed activity of **2** and **3** cannot be excluded, however, and further studies are needed.

Involvement in neuronal development and survival as well as dysregulation in Alzheimer’s and Parkinson’s disease support therapeutic potential of Nurr1 in neurodegenerative pathologies. Therefore, interest in Nurr1 ligand development has dramatically increased and several potent agonists have been developed^35^. Here, we explored whether Nurr1, which can act in different oligomeric states^3^ including RXR heterodimers, could be selectively modulated via dual Nurr1/RXR activation. We identified the natural product valerenic acid and a previously reported mimetic **2**^32^ as dual agonists and enhanced dual potency by aromatization of **2** to **3**. In line with our hypothesis, the dual Nurr1/RXR ligands **2** and **3** had a different impact on Nurr1 dimers than classical agonists and only disrupted the Nurr1 homodimer while leaving the heterodimer intact. In cellular setting, this translated into selective activation of the Nurr1:RXR heterodimer response element DR5. Moreover, changes in neuronal gene expression in response to treatment with **2** or **3** differed from classical Nurr1 agonist effects as only a subset of agonist-induced genes was upregulated. This could be explained in part by the predicted presence of DR5 in the promoter regions of the evaluated genes. Though further research on the mechanisms of the observed effects and structural optimization of the dual ligands is needed, these results suggest that oligomer-specific Nurr1 activation may open an avenue to gene-selective modulation. Our results hence provide initial proof-of-concept for promising effects of dual Nurr1/RXR agonism and further evaluation of this concept of Nurr1 modulation is warranted.

## Supplementary Material

**Associated content**Supporting Information (pdf) containing Figures S1−S5, synthetic procedures and analytical characterization data for compounds **2−5**, methods for in vitro characterization.

Supporting info.

## Figures and Tables

**Figure 1:**
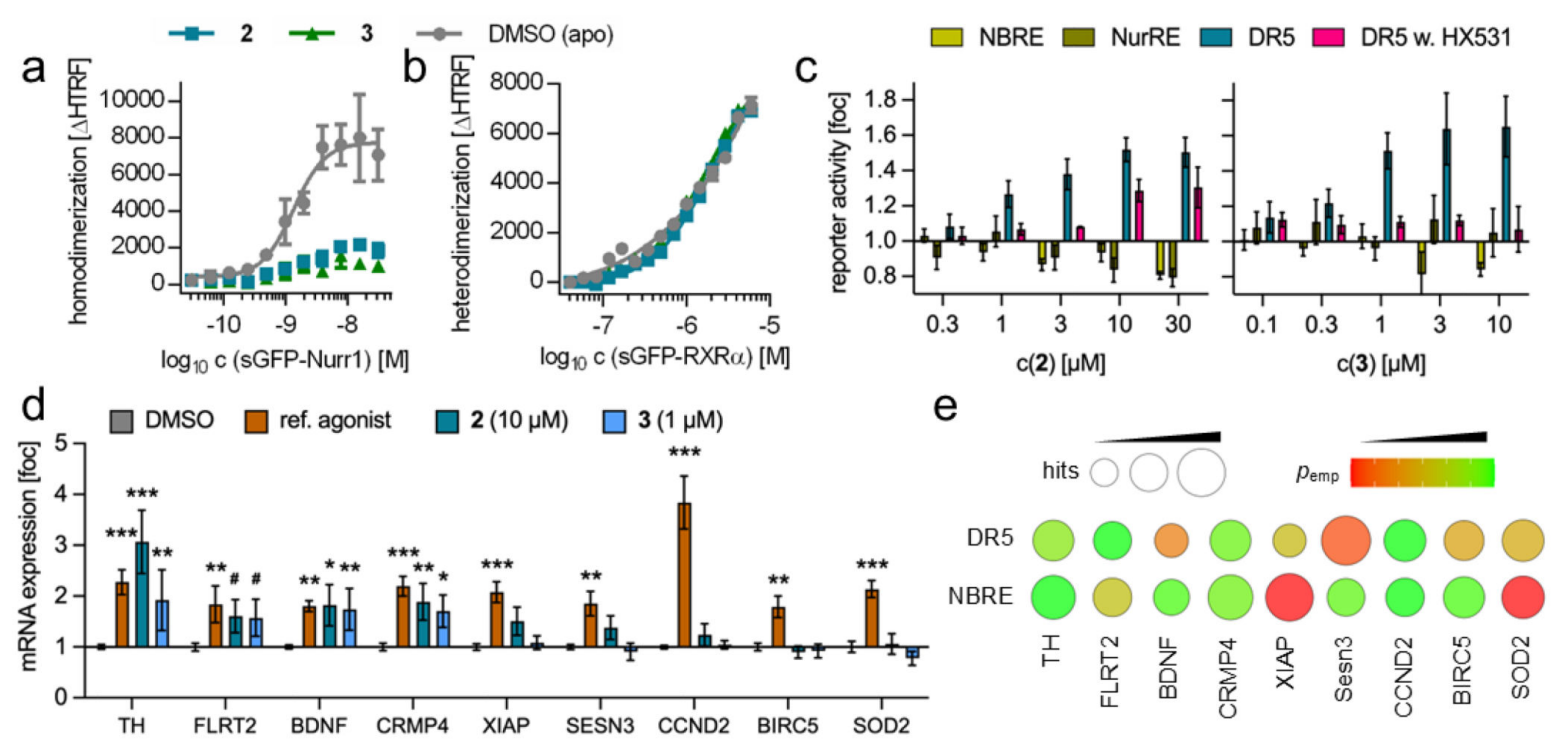
Dual Nurr1/RXR agonists mediated selective Nurr1 modulation. (a) Impact of **2** and **3** (30 μM each) on Nurr1 homodimer formation in a homogenous time-resolved fluorescence resonance energy transfer (HTRF) based assay with Tb^3+^-cryptate labeled and sGFP-labeled Nurr1 LBD proteins. Data are the mean±SD, n=3. (b) Impact of **2** and **3** (30 μM each) on Nurr1 heterodimer formation in an HTRF based assay with Tb^3+^-cryptate labeled Nurr1 LBD and sGFP-labeled RXRα LBD proteins. Data are the mean±SD, n=3. (c) Effects of **2** and **3** on full-length Nurr1 activity on the monomer (NBRE), homodimer (NurRE) and heterodimer (DR5; in absence or presence of the RXR antagonist HX531^37^ (1 μM)) response elements in uniform reporter gene assays. **2** and **3** activated exclusively the Nurr1:RXR heterodimer (EC_50_ 1.0 μM (**2**); 0.6 μM (**3**)). Data are the mean±S.E.M., n≥3 (d) Effects of **2** and **3** on gene expression in rat dopaminergic neurons (N27). The Nurr1 agonist 32 (1 μM) from ^39^ was used as reference agonist. Data are the mean±SD, n=5, *** p≤0.001, ** p≤0.01, * p≤0.05, # p≤0.1 (2way ANOVA vs. DMSO control). (e) Promoter analysis of the studied genes for the presence of NBRE and DR5 with JASPAR^40^ showing the detected frequency (circle size) and median empirical probability (color).

**Scheme 1:**
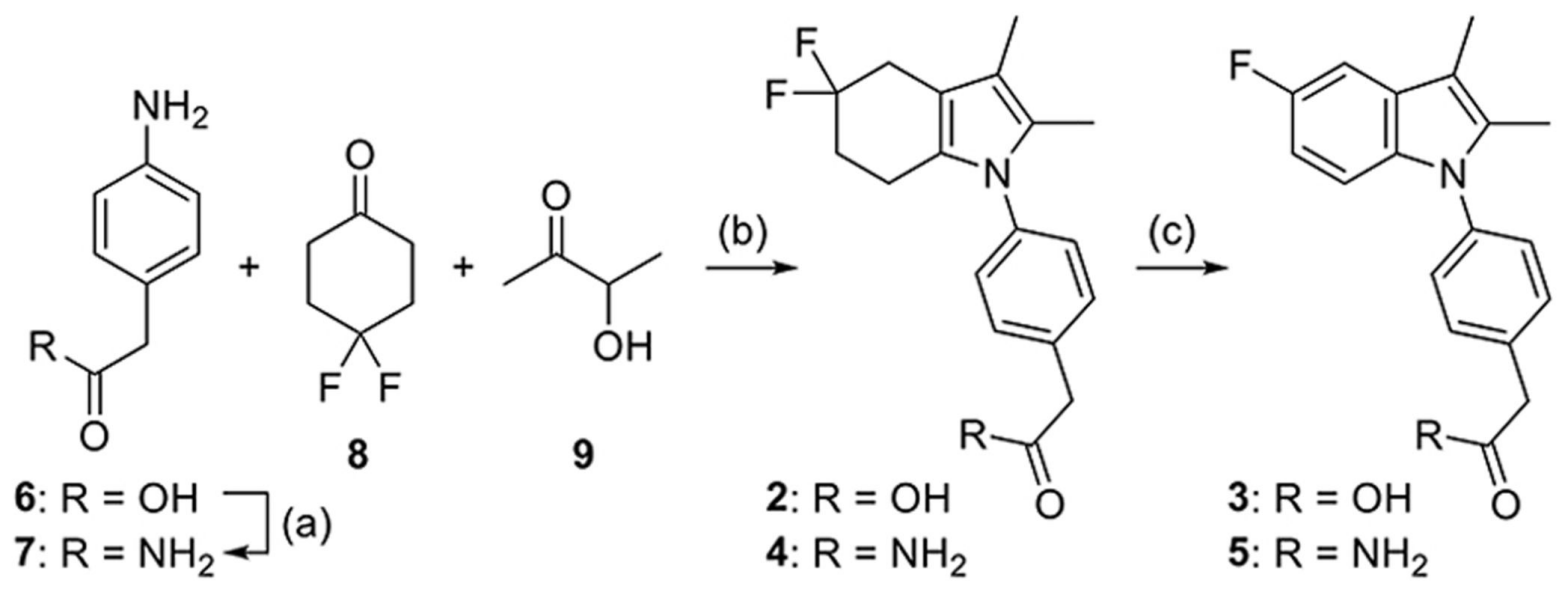
Synthesis of compounds **2** – **5**. Reagents & Conditions: (a) H_2_SO_4_, MeOH, 65 °C, 1 h; then NH_3_, NaCl (aq. sat.), RT, overnight, 49% over 2 steps. (b) TFA, toluene, 100 °C, 2 h, 8–15%. (c) TFA, toluene, RT, 96 h, 3–5% over 2 steps.

**Table 1:** Dual Nurr1/RXR agonist profiles of valerenic acid (**1**) and synthetic mimetics.

ID	structure	EC_50_(Nurr1) (max. act.) ^a^	Kd(Nurr1 LBD) ^b^	EC_50_(RXR) (max. act.) ^a^	Kd(RXRα LBD) ^b^
**1**	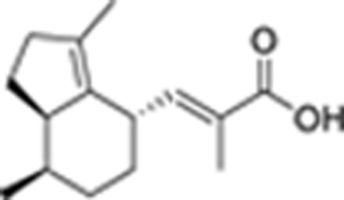	3±1 μM (1.3±0.1-fold)	not determined	α: 27±3 μM (9±1-fold) β: 5.2±0.4 μM (69±1-fold) γ: 43±1 μM (4±1-fold)	not determined
**2**	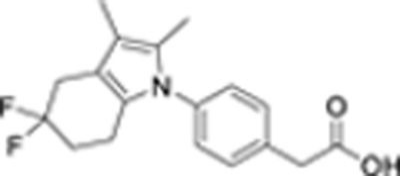	2.6±0.4 μM (3.3±0.1-fold)	0.6 μM	α: 0.97±0.07 μM (69±1-fold) β: 4.5±0.8 μM (121±6-fold) γ: 3.4±0.4 μM (44±1-fold)	1.1 μM
**3**	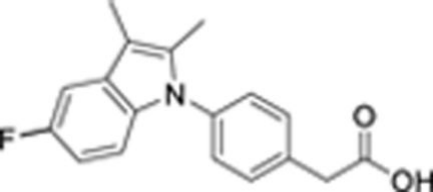	2.2±0.9 μM (4.3±0.5-fold)	0.6 μM	α: 0.4±0.1 μM (83±9-fold) β: 0.6±0.1 μM (103±6-fold) γ: 1.7±0.2 μM (67±4-fold)	0.2 μM
**4**	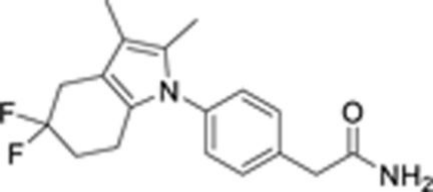	86±9% remain. Nurr1 activity (30 μM)	no binding detected	α: inactive (30 μM) β: inactive (30 μM) γ: inactive (30 μM)	no binding detected
**5**	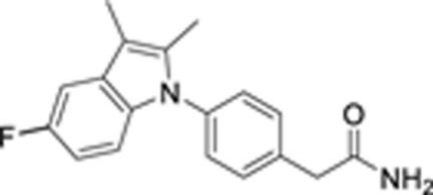	85±7% remain. Nurr1 activity (30 μM)	no binding detected	α: inactive (30 μM) β: inactive (30 μM) γ: inactive (30 μM)	no binding detected

a Nurr1 and RXR modulation were determined in uniform Gal4-hybrid reporter gene assays. Data are the mean±S.E.M., n≥3.

b Binding to the recombinant Nurr1 and RXRα LBD proteins was determined by isothermal titration calorimetry (ITC). Binding isotherms are shown in Figure S2.

## Abbreviations

BDNF: brain-derived neurotrophic factor
BIRC5: baculoviral inhibitor of apoptosis repeat-containing 5
CCND2: cyclin D2
CRMP4: collapsin response mediator protein 4
DR5: direct repeat 5
FLRT2: fibronectin leucine rich transmembrane protein 2
HTRF: homogeneous time-resolved fluorescence resonance energy transfer
ITC: isothermal titration calorimetry
LBD: ligand binding domain
NBRE: nerve growth factor-inducible-β-binding response element
Nurr1: nuclear receptor related 1
NurRE: Nur response element
RE: response element
RXR: retinoid X receptor
SESN3: sestrin 3
SOD2: superoxide dismutase 2
TH: tyrosine hydroxylase
XIAP: X-linked inhibitor of apoptosis protein
