# Supplementary material for: Dual Nurr1/RXR agonism of valerenic acid and synthetic mimetics enables dimer-selective Nurr1 modulation
Source: ACS Med Chem Lett. Author manuscript; Available in PMC 2026 Jan 7. (PMC7618597; doi:10.1021/acsmedchemlett.5c00572)
Supplement: Supporting info. [file EMS211515-supplement-Supporting_info_.pdf]

– Supporting Information –

Dual Nurr1/RXR Agonism of Valerenic Acid and Synthetic Mimetics Enables Dimer-Selective Nurr1 Modulation

Katharina Scholz[1], Úrsula López-García[1], Romy Busch[1], Julian A. Marschner[1], Daniel Merk[1]*

[1] Ludwig-Maximilians-Universität München, Department of Pharmacy, 81377 Munich, Germany

* daniel.merk@cup.lmu.de

Table of Contents

Figures S1 – S5 .... S2

Methods S5

NMR spectra of **2–5** S11

Supplementary References S19

Supplementary Figures

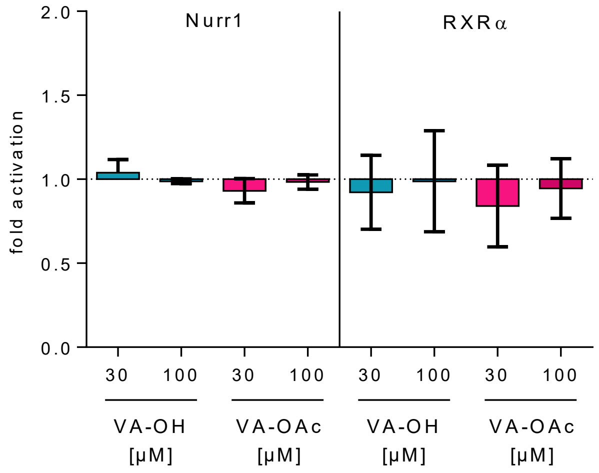

Figure S1. Natural valerenic acid derivatives hydroxy- (VA-OH) and acetoxy- (VA-OAc) valerenic acid exhibited no activity on Nurr1 and RXR in Gal4 hybrid reporter gene assays. Data are the mean±SD fold reporter activation vs. DMSO ctrl, n=3.

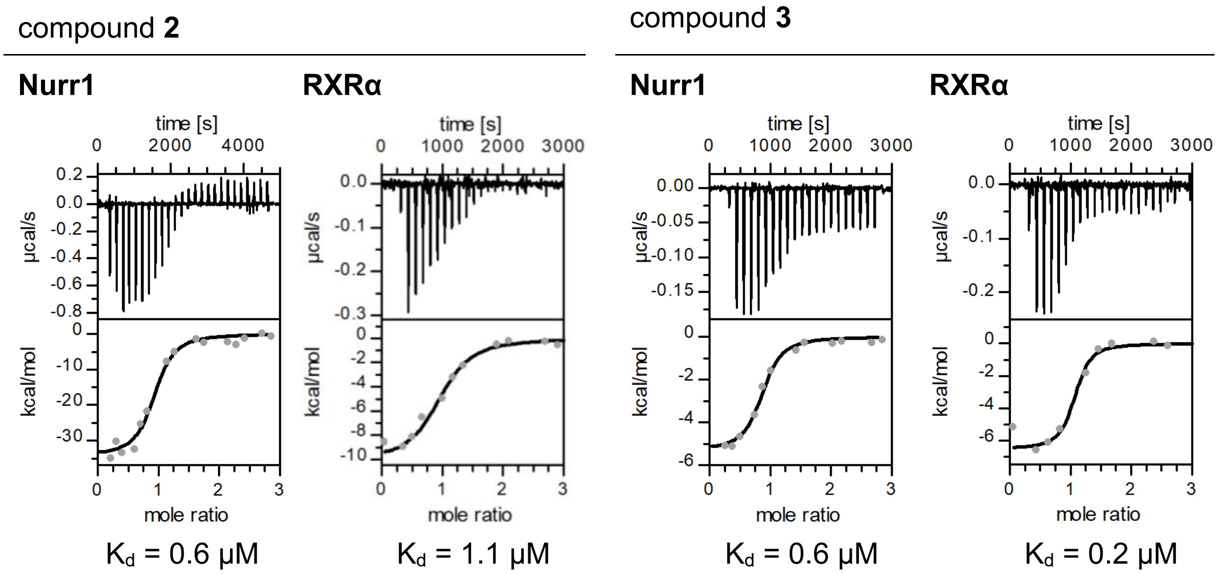

Figure S2. Isothermal titration calorimetry (ITC) demonstrated binding of **2** and **3** to the ligand binding domains of both Nurr1 and RXRα. The upper panels show the binding isotherms, the lower panels show the fitting of the heat of binding.

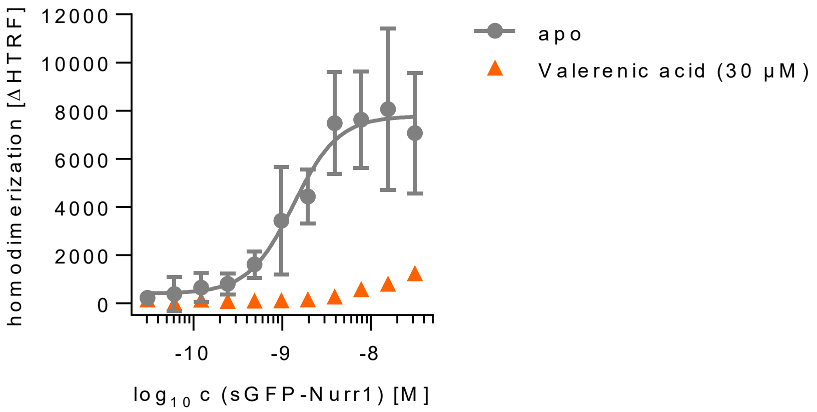

Figure S3. Impact of valerenic acid (**1**, 30 µM) on Nurr1 homodimer formation in a homogenous time-resolved fluorescence resonance energy transfer (HTRF) based assay with Tb^{3+}-cryptate labeled Nurr1 LBD and sGFP-labeled Nurr1 LBD proteins. Data are the mean±SD, n=3.

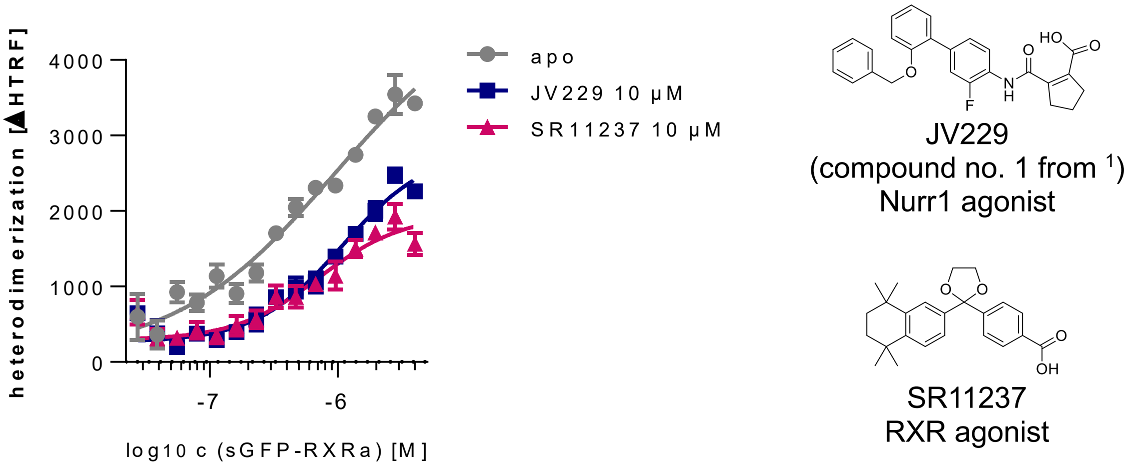

Figure S4. Impact of SR11237 (10 µM) and JV229 (10 µM) on Nurr1 heterodimer formation in a homogenous time-resolved fluorescence resonance energy transfer (HTRF) based assay with Tb^{3+}-cryptate labeled RXRα LBD and sGFP-labeled Nurr1 LBD proteins. Data are the mean±SD, n=3.

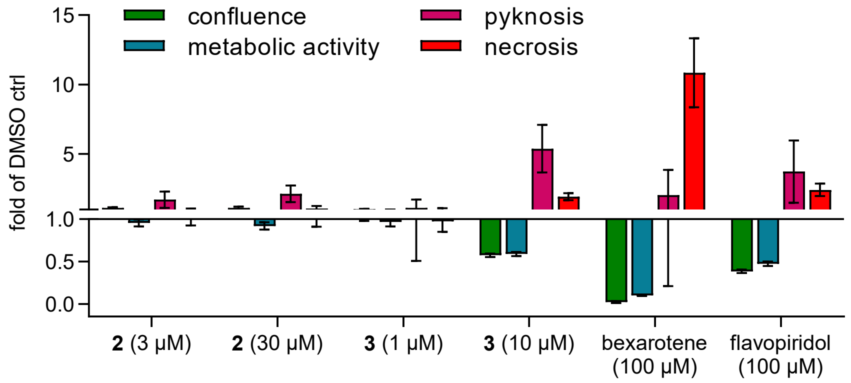

Figure S5. **2** exhibited no toxicity up to 30 µM, **3** exhibited toxic effects starting at 10 µM in a multiplex toxicity assay in HEK293T cells. Data are the mean±SD; n=4; bexarotene (100 µM) and flavopiridol (100 µM) as positive control.

Methods

Chemistry

General. All chemicals and solvents were of reagent grade and used without further purification unless otherwise specified. Other solvents, especially for work-up procedures, were of reagent grade or purified by distillation (*i*-hexane, EtOAc, EtOH). All reported temperatures are heating block temperatures. Reactions were monitored by thin layer chromatography (TLC) on TLC Silica gel 60 F_{254} aluminium sheets by Merck and visualized under ultraviolet light (254 nm). Purification of compounds by column chromatography was performed on a PuriFlash® XS520Plus system from Advion Interchim using high performance spherical silica columns (SIHP, 15/30/50 µm) and a gradient of *i*-hexane to EtOAc, reversed-phase column chromatography was performed on a PuriFlash® 5.250 system from Advion Interchim using C18HP columns (15 µm) and a gradient from 10% acetonitrile in H_2O (purified by a Milli-Q® 7000 Ultrapure Water System) to 100% acetonitrile (HPLC gradient grade). High Resolution mass spectrometry (HRMS) analyses were performed on a Thermo Exploris 120 (Thermo Fisher Scientific) using electrospray ionization (ESI) or atmospheric pressure chemical ionization (APCI). ^{1}H, $^{13}C\{^{1}H\}$, and $^{19}F\{^{1}H\}$ nuclear magnetic resonance (NMR) spectra were recorded on Bruker Avance III HD 400 MHz or 500 MHz spectrometers. Chemical shifts (δ) are reported in parts per million (ppm). The NMR spectra were calibrated using the proton or carbon signals of residual non-deuterated solvent peaks (2.05 and 29.84 ppm for acetone-d_6). Signal multiplicity is reported as follows: s, singlet; bs, broad singlet; d, doublet; dd, doublet of doublets; t, triplet; td, triplet of doublets; m, multiplet. Quantitative ^{1}H NMR spectra (qHNMR) were acquired according to a method described by Pauli et al.[2] with internal calibration. The qHNMR measurements were carried out under conditions allowing complete relaxation to assure the exact determination of peak area ratios. Used internal standards were dimethyl terephthalate (lot no. #BCBT9974, purity 99.95%) or maleic acid (lot no. #BCCK2148, purity 99.93%). All final compounds to be tested in the biological evaluation had a purity of >95% according to qHNMR. 2-(4-aminophenyl)acetamide (**7**) was obtained according to a literature procedure[3].

2-(4-(5,5-Difluoro-2,3-dimethyl-4,5,6,7-tetrahydro-1*H*-indol-1-yl)phenyl)acetic acid (2). 2-(4-aminophenyl)acetic acid (156 mg, 1.00 mmol, 1.00 eq.), acetoin (110 mg, 1.25 mmol, 1.25 eq.) and 4,4-difluorocyclohexanone (134 mg, 1.00 mmol, 1.00 eq.) were combined in a pressure tube under Argon atmosphere and trifluoroacetic acid/toluene (0.8%, 10 mL) was added. The pressure tube was closed and stirred at 100 °C for 2 h. Water was added and the aqueous layer extracted with ethyl acetate (3 x), the combined organic layers were dried over $MgSO_4$, filtered and the solvent removed under reduced pressure. The crude product was purified by normal phase and reverse phase column chromatography giving **2** (47 mg, 0.15 mmol, 15%) as light-beige solid. ^{1}H NMR (400 MHz, acetone-d_6) δ 7.49–7.41 (m, 2H), 7.23–7.15 (m, 2H), 3.72 (s, 2H), 2.92 (t, J = 14.4 Hz, 2H), 2.51 (t, J = 6.6 Hz, 2H), 2.21–2.08 (m, 2H), 1.95 (s, 3H), 1.91 (s, 3H) ppm. $^{13}C\{^{1}H\}$ NMR (101 MHz, acetone-d_6) δ 172.5, 138.2, 135.2, 131.1, 128.4, 125.7, 125.6 (t, J = 240.6 Hz), 124.3, 113.2 (t, J = 6.0 Hz), 113.0, 40.7, 32.6 (t, J = 26.7 Hz), 32.0 (t, J = 25.2 Hz), 20.8 (t, J = 5.6 Hz), 10.6, 9.1 ppm. $^{19}F\{^{1}H\}$ NMR (376 MHz, acetone-d_6) δ -97.76 ppm. HRMS (ESI+) *m/z* calculated for $C_{18}H_{20}F_2NO_2$ $[M+H]^+$: 320.1457, found: 320.1454.

2-(4-(5-Fluoro-2,3-dimethyl-1*H*-indol-1-yl)phenyl)acetic acid (3). 2-(4-aminophenyl)acetic acid (78 mg, 0.50 mmol, 1.00 eq.), acetoin (55 mg, 0.63 mmol, 1.25 eq.) and 4,4-difluorocyclohexanone (67 mg, 0.50 mmol, 1.00 eq.) were combined in a pressure tube under Argon atmosphere and trifluoroacetic acid/toluene (0.8%, 10 mL) was added. The pressure tube was closed and stirred at 100 °C for 2 h. The mixture was then transferred to a round bottom flask and stirred for 96 h under air. Water was added and the aqueous layer extracted

with ethyl acetate (3 x), the combined organic layers dried over $MgSO_4$, filtered and the solvent removed under reduced pressure. The crude product was purified by normal phase and reverse phase column chromatography giving **3** (6.9 mg, 0.023 mmol, 5%) as light-beige solid. ^{1}H NMR (500 MHz, acetone-*d*$_6$) δ 7.55 (d, *J* = 8.0 Hz, 2H), 7.35 (d, *J* = 8.0 Hz, 2H), 7.18 (dd, *J* = 9.7, 2.5 Hz, 1H), 6.99 (dd, *J* = 8.8, 4.4 Hz, 1H), 6.81 (td, *J* = 9.1, 2.6 Hz, 1H), 3.77 (s, 2H), 2.26 (s, 3H), 2.22 (s, 3H) ppm. ^{13}C{^{1}H} NMR (126 MHz, acetone-*d*$_6$) δ 172.7, 158.9 (d, *J* = 232.7 Hz), 137.5, 135.8, 135.7, 134.9, 131.6, 130.3 (d, *J* = 9.4 Hz), 128.5, 111.1 (d, *J* = 9.4 Hz), 109.5 (d, *J* = 25.9 Hz), 108.6 (d, *J* = 4.2 Hz), 103.5 (d, *J* = 23.5 Hz), 41.0, 11.1, 8.9 ppm. ^{19}F{^{1}H} NMR (376 MHz, acetone-*d*$_6$) δ -127.51 ppm. HRMS (ESI−) *m/z* calculated for $C_{36}H_{31}F_2N_2O_4$ [2 M−H]$^-$: 593.2257, found: 593.2251.

2-(4-(5,5-Difluoro-2,3-dimethyl-4,5,6,7-tetrahydro-1*H*-indol-1-yl)phenyl)acetamide (4). 2-(4-aminophenyl)acetamide (50 mg, 0.33 mmol, 1.00 eq.), acetoin (37 mg, 0.42 mmol, 1.25 eq.) and 4,4-difluorocyclohexanone (45 mg, 0.33 mmol, 1.00 eq.) were combined in a pressure tube under Argon atmosphere and trifluoroacetic acid/toluene (0.8%, 10 mL) was added. The pressure tube was closed and stirred at 100 °C for 2 h. Water was added and the aqueous layer extracted with ethyl acetate (3 x), the combined organic layers dried over $MgSO_4$, filtered and the solvent removed under reduced pressure. The crude product was purified by normal phase and reverse phase column chromatography giving **4** (8 mg, 0.03 mmol, 8%) as beige solid. ^{1}H NMR (500 MHz, acetone-*d*$_6$) δ 7.48–7.42 (m, 2H), 7.19–7.13 (m, 2H), 6.91 (s, 1H), 6.28 (s, 1H), 3.57 (s, 2H), 2.92 (t, *J* = 14.4 Hz, 2H), 2.51 (t, *J* = 6.6 Hz, 2H), 2.20–2.08 (m, 2H), 1.94 (s, 3H), 1.91 (s, 3H) ppm. ^{13}C{^{1}H} NMR (126 MHz, acetone-*d*$_6$) δ 172.6, 138.0, 136.7, 130.9, 128.3, 125.7, 125.6 (t, *J* = 240.4 Hz), 124.2, 113.1 (t, *J* = 5.9 Hz), 112.9, 42.8, 32.6 (t, *J* = 26.8 Hz), 32.0 (t, *J* = 25.4 Hz), 20.7 (t, *J* = 5.9 Hz), 10.6, 9.1 ppm. ^{19}F{^{1}H} NMR (376 MHz, acetone-*d*$_6$) δ -97.75 ppm. HRMS (ESI−) *m/z* calculated for $C_{18}H_{19}F_2N_2O$ [M−H]$^-$: 317.1471, found: 317.1471.

2-(4-(5-Fluoro-2,3-dimethyl-1*H*-indol-1-yl)phenyl)acetamide (5). 2-(4-aminophenyl)acetamide (50 mg, 0.33 mmol, 1.00 eq.), acetoin (37 mg, 0.42 mmol, 1.25 eq.) and 4,4-difluorocyclohexanone (45 mg, 0.33 mmol, 1.00 eq.) were combined in a pressure tube under Argon atmosphere and trifluoroacetic acid/toluene (0.8%, 10 mL) was added. The pressure tube was closed and stirred at 100 °C for 2 h. The mixture was then transferred to a round bottom flask and stirred for 96 h under air. Water was added and the aqueous layer extracted with ethyl acetate (3 x), the combined organic layers dried over $MgSO_4$, filtered and the solvent removed under reduced pressure. The crude product was purified by normal phase and reverse phase column chromatography giving **5** (2.8 mg, 0.0095 mmol, 3%) as light-beige solid. ^{1}H NMR (400 MHz, acetone-*d*$_6$) δ 7.60–7.53 (m, 2H), 7.38–7.30 (m, 2H), 7.20 (dd, *J* = 9.7, 2.5 Hz, 1H), 7.00 (dd, *J* = 8.8, 4.4 Hz, 1H), 6.96 (bs, 1H), 6.82 (td, *J* = 9.1, 2.6 Hz, 1H), 6.32 (bs, 1H), 3.64 (s, 2H), 2.27 (s, 3H), 2.24 (s, 3H) ppm. ^{13}C{^{1}H} NMR (126 MHz, acetone-*d*$_6$) δ 171.7, 158.9, 157.0, 136.3 (d, *J* = 12.7 Hz), 134.9, 134.0, 130.4, 129.4 (d, *J* = 9.9 Hz), 127.6, 110.3 (d, *J* = 9.9 Hz), 108.5 (d, *J* = 26.4 Hz), 107.6 (d, *J* = 4.7 Hz), 102.5 (d, *J* = 23.5 Hz), 41.9, 10.2, 8.0 ppm. ^{19}F{^{1}H} NMR (376 MHz, acetone-*d*$_6$) δ -127.55 ppm. HRMS (APCI+) *m/z* calculated for $C_{18}H_{18}FN_2O$ [M+H]$^+$: 297.1398, found: 297.1394.

In vitro characterization

Hybrid reporter gene assays. Nurr1 and RXR modulation was determined in Gal4 hybrid reporter gene assays in transiently transfected HEK293T cells (German Collection of Microorganisms and Cell Culture GmbH, DSMZ) using pFR-Luc (Stratagene, La Jolla, CA, USA; reporter), pRL-SV40 (Promega, Madison, WI, USA; internal control), and the previously described[4] hybrid receptor plasmids pFA-CMV-hNurr1-LBD, pFA-CMV-hRXRα-LBD, pFA-CMV-hRXRβ-LBD, pFA-CMV-hRXRγ-LBD. HEK293T cells were cultured in Dulbecco's

modified Eagle's medium (DMEM), high glucose supplemented with 10% fetal calf serum (FCS), sodium pyruvate (1 mM), penicillin (100 U/mL), and streptomycin (100 µg/mL) at 37 °C and 5% CO_2 and seeded in 96-well plates (3×10^4 cells/well). After 24 h, the medium was changed to Opti-MEM without supplements, and the cells were transiently transfected using Lipofectamine LTX reagent (Invitrogen) according to the manufacturer's protocol. Five hours after transfection, the cells were incubated with the test compounds in Opti-MEM supplemented with penicillin (100 U/mL), streptomycin (100 µg/mL), and 0.1% DMSO for 15 h before luciferase activity was measured using the Dual-Glo Luciferase Assay System (Promega) according to the manufacturer's protocol on a Tecan Spark luminometer (Tecan Deutschland GmbH, Germany). Firefly luminescence was divided by Renilla luminescence and multiplied by 1000, resulting in relative light units (RLU) to normalize for transfection efficiency and cell growth. Fold activation was obtained by dividing the mean RLU of a sample by the mean RLU of the untreated control. All samples were tested in at least three biologically independent experiments in duplicates. For dose–response curve fitting and calculation of EC_{50} values, the equation "[Agonist] vs. response – Variable slope (four parameters)" was used in GraphPad Prism (version 5.04, GraphPad Software, La Jolla, CA, USA). Fluvastatin (10 µM, NR4A) and bexarotene (1 µM, NR2B) served as the positive control.

Reporter gene assays for full-length human Nurr1 and RXR. Modulation of full-length human Nurr1 (NR4A2) was studied using pRL-SV40 (1.5 ng/well reporter plasmid) and the previously described[4] reporter plasmids pFR-Luc-NBRE (100 ng/well reporter plasmid), pFR-Luc-NurRE (50 ng/well reporter plasmid) or pFR-Luc-DR5 (50 ng/well reporter plasmid). The full-length human nuclear receptor Nurr1 encoded by pcDNA3.1-hNurr1-NE (gift from Shu Leong Ho, Addgene plasmid #102363), and full length human RXRα encoded by pSG5-hRXR were mildly overexpressed (1 ng/well receptor plasmid each). HEK293T cells were cultured in DMEM, high glucose supplemented with 10% FCS, sodium pyruvate (1 mM), penicillin (100 U/mL), and streptomycin (100 µg/mL) at 37 °C and 5% CO_2 and seeded in 96-well plates (3×10^4 cells/well). After 24 h, the medium was changed to Opti-MEM without supplements, and the cells were transiently transfected using Lipofectamine LTX reagent (Invitrogen) according to the manufacturer's protocol. Five hours after transfection, the cells were incubated with the test compounds in Opti-MEM supplemented with penicillin (100 U/mL), streptomycin (100 µg/mL), and 0.1% DMSO for 15 h before luciferase activity was measured using the Dual-Glo Luciferase Assay System (Promega) according to the manufacturer's protocol on a Tecan Spark luminometer (Tecan Deutschland GmbH, Germany). Firefly luminescence was divided by Renilla luminescence and multiplied by 1000, resulting in relative light units (RLU) to normalize for transfection efficiency and cell growth. Fold activation was obtained by dividing the mean RLU of a sample by the mean RLU of the untreated control. All samples were tested in at least three biologically independent experiments in duplicates. For dose–response curve fitting and calculation of EC_{50} values, the equation "[Agonist] vs. response – Variable slope (four parameters)" was used in GraphPad Prism (version 5.04, GraphPad Software, La Jolla, CA, USA). Amodiaquin (100 µM) served as positive control.

Isothermal titration calorimetry. ITC experiments for ligand binding to Nurr1 and RXR were conducted on an Affinity ITC instrument (TA Instruments, New Castle, DE) at 25 °C with a stirring rate of 75 rpm using recombinantly expressed ligand binding domain proteins as described previously[5,6]. The respective LBD protein (12–25 µM) in buffer (20 mM Tris pH 7.5, 100 mM NaCl, 5% glycerol) containing 2-4% DMSO was titrated with the test compounds (100-150 µM in the same buffer containing 2-4% DMSO) in 21–26 injections (1 × 1 µL and 20 × 4 to 25 × 4 µL) with an injection interval of 120 s. As control experiments, the test compounds were titrated to the buffer, and the buffer was titrated to the protein under otherwise identical conditions. The heat rates of the compound-Nurr1 LBD titrations were analyzed using NanoAnalyze software (TA Instruments, New Castle, DE) with an independent binding model.

Nurr1 dimerization assays (HTRF). Modulation of Nurr1 LBD homodimerization and heterodimerization of the LDBs of Nurr1 and RXRα was investigated by HTRF as reported previously[4]. Nurr1 LBD or RXRα LBD proteins labeled with sGFP were titrated against a fixed concentration of Tb^{3+}-cryptate as streptavidin conjugate (Tb-SA) (610SATLF, Revvity) coupled to biotinylated Nurr1 LBD protein in the presence of test compounds at 30 µM or absence of compound as a negative control (apo). Assay solutions were prepared in buffer containing 25 mM HEPES pH 7.5, 150 mM KF, 5% (m/v) glycerol, 5 mM DTT, 0.1% (m/v) CHAPS and 1% DMSO. The FRET donor complex was formed from biotinylated Nurr1 LBD (final concentration 0.188 nM) and Tb-SA (0.375 nM) and was kept constant. For the homodimerization assay, the FRET acceptor consisted of sGFP-Nurr1 LBD, which was titrated from 0.5 µM, while free sGFP was added to keep the total GFP content stable at 0.5 µM. For the heterodimerization assay, the FRET acceptor sGFP-RXRα LBD was titrated from 6 µM, while free sGFP was added to keep a constant concentration of GFP at 6 µM. Samples were equilibrated at room temperature for 2 h and fluorescence intensities (FI) after excitation at 340 nm were recorded at 520 nm for sGFP acceptor fluorescence and at 620 for Tb-SA donor fluorescence on a SPARK plate reader (Tecan Group Ltd.). FI520 was divided by FI620 and multiplied by 10,000 to give a dimensionless HTRF signal. For dose-response curve fitting, the equation "[Agonist] versus response–variable slope (four parameters)" was used in GraphPad Prism (version 9.5.1, GraphPad Software, La Jolla, CA).

Evaluation of Nurr1-regulated gene expression. N27 rat dopaminergic neural cells (SCC048, Sigma-Aldrich, Darmstadt, Germany) were cultured in RPMI 1640 medium (Gibco, Thermo Fisher Scientific, Waltham, MA, USA) supplemented with 10% FCS, penicillin (100 U/mL), and streptomycin (100 µg/mL) at 37 °C and 5% and seeded in 12-well plates (3×10^5 cells/well). After 7 h, the medium was changed to RPMI 1640 medium (Gibco, Thermo Fisher Scientific) supplemented with 0.2% FCS, penicillin (100 U/mL), and streptomycin (100 µg/mL), and the cells were incubated for another 22 h, before the medium was changed again to RPMI 1640 medium (Gibco, Thermo Fisher Scientific) supplemented with 0.2% FCS, penicillin (100 U/mL), and streptomycin (100 µg/mL), additionally containing **2**, **3** or the Nurr1 agonist 32 from [7] in 0.1% DMSO or 0.1% DMSO alone. After 21 h of incubation, the medium was removed, cells were washed with phosphate-buffered saline (PBS), and after full aspiration of residual liquids immediately frozen at -80 °C until further processing. Total RNA was isolated using peqGOLD Total RNA Kit (VWR International, Darmstadt, Germany) following the manufacturer's instructions. RNA concentration and purity were assessed using a NanoDrop One UV-vis spectrophotometer (Thermo Fisher Scientific) at 260/280 nm. Right before reverse transcription (RT), RNA was linearized at a concentration of 133 ng/µL at 65 °C for 10 min and then immediately incubated on ice for at least 1 min. Reverse transcription was performed using 2 µg of total RNA, 20 U Recombinant RNasin Ribonuclease Inhibitor (Promega, Mannheim, Germany), 100 U SuperScript IV Reverse Transcriptase including 5× First Strand Buffer and 0.1 M dithiothreitol (Thermo Fisher Scientific), 3.75 ng of linear acrylamide, 625 ng of random hexamer primers (Merck, Darmstadt, Germany), and 11.25 nmol of deoxynucleoside triphosphate mix (2.8 nmol each ATP, TTP, CTP, GTP; Thermo Fisher Scientific) at a volume of 22.45 µL at 50 °C for 10 min and 80 °C for 10 min using a Thermal cycler XT96 (VWR International). A quantitative polymerase chain reaction (qPCR) was conducted using a qTOWERiris (Analytik Jena, Jena, Germany) and a SYBR green-based detection method. 0.2 µL of prepared cDNA was added to 6 pmol each of forward and reverse primer, 0.8 U Taq DNA Polymerase (New England Biolabs, Ipswich, MA, USA), 40 ppm SYBR Green I (Sigma-Aldrich), 15 nmol of deoxynucleoside triphosphate mix (as indicated above), 60 nmol of $MgCl_2$, 4 µg of bovine serum albumin (Thermo Fisher Scientific), 20% BioStab PCR Optimizer II (Merck, Darmstadt, Germany), and 10% Taq buffer without detergents (Thermo Fisher Scientific), topped up to a final volume of 20 µL with ddH_2O. Samples underwent 40 cycles of 15 s denaturation at 95 °C, 15 s of primer annealing at primer-specific temperatures

and 20 s of elongation at 68 °C. PCR product specificity was evaluated using a melting curve analysis ranging from 65 to 95 °C. Nurr1 target gene expression was normalized to rGAPDH mRNA expression per sample using the ΔCt-method. The following primers and annealing temperatures were used: rGAPDH (59.4 °C): 5'-CAG CCG CAT CTT CTT GTG C-3' (fwd), 5'-AAC TTG CCG TGG GTA GAG TC-3' (rev); rTH (59.4 °C): 5'-TGG GGA GCT GAA GGC TTA TG-3' (fwd), 5'-AGA GAA TGG GCG CTG GAT AC-3' (rev); rFLRT2 (59.0 °C): 5'-AAG GAG ACA AGG CTA CCA GAT TAC-3' (fwd), 5'-GCA AAG CGT GAT GCC AAG TA-3' (rev); rSESN3 (62.4 °C): 5'-TCG GCC AAC TAC CTG CTC TG-3' (fwd), 5'-CGT GTT TGC TTG GAC AAC TTC CT-3' (rev); rCRMP4 (58.0 °C): 5'-TGT CCT ACC AGG GCA AGA A-3' (fwd), 5'-ATC AGA TTG TCT CCA ATT TGC TTT A-3' (rev); rBDNF (58.0 °C): 5'-AGT CTA GAA CCT TGG GGA CC-3' (fwd), 5'-GCC TTC ATG CAA CCG AAG TA-3' (rev); rBIRC5 (59.4 °C): 5'-TCC ACT GCC CTA CCG AGA AT-3' (fwd), 5'-AGG GGA GTG CTT CCT ATG CT-3' (rev); rCCND2 (59.0 °C): 5'-CAA GTT TGC CAT GTA CCC GC-3' (fwd), 5'-GCT TTG AGA CAA TCC ACA TCG G-3' (rev); rXIAP (61.1 °C): 5'-TCA CTT GGG GAA TCT GTG GTA AG-3' (fwd), 5'-TCC CAG ATG TTT GGA GCT TTT CT-3' (rev); rSOD2 (59.4 °C): 5'-CGG GGG CCA TAT CAA TCA CA-3' (fwd), 5'-TCC AGC AAC TCT CCT TTG GG-3' (rev).

Multiplex toxicity assay. HEK293T cells were grown in DMEM high glucose, supplemented with 10% FCS, sodium pyruvate (1 mM), penicillin (100 U/mL), and streptomycin (100 µg/mL) at 37 °C and 5% CO_2. The day before experiment, cells were seeded in 96-well plates (2×10^4 cells per well) in culture medium with reduced serum content (0.2%). The next day, medium was changed, maintaining the low serum content and additionally containing 0.1% DMSO with **2** (1 or 10 µM) or **3** (3 or 30 µM), 0.1% DMSO with bexarotene (100 µM) or flavopiridol (100 µM) as positive controls, or 0.1% DMSO alone as untreated control. Each sample was prepared in 4 biologically independent replicates. After incubation for 24 h, the medium was changed to 90 µL culture medium without phenol red (0.2% FCS) and 10 µL Cell Counting Kit-8 solution (CCK-8, MedChem Express #HY-K0301), and absorbance was measured after 2 h incubation at 450 nm on a Tecan Spark Cyto (Tecan Group AG) to assess metabolic activity of the cells. Thereafter, Hoechst33342 (10 µM, #ab228551, Abcam Limited, Cambridge, UK) and Live-or-Dye Nuc-Fix Red (0.05×, Biotium, Inc., Fremont, CA, 1691 USA) were added and incubated for 30 min to detect pyknosis and cellular necrosis, respectively. After incubation, a total of 3 fluorescence images per well at 10× magnification was taken to detect Hoechst33342-positive cell nuclei (Ex: 381−400 nm, Em: 414−450 nm) and Live-or-Dyepositive cells (Ex: 543−566 nm, Em: 580−611 nm), respectively, using a Tecan Spark Cyto (Tecan Group AG). Necrotic cells were counted using CellProfiler (Version 4.2.6). Reference readings for background correction and detection of autofluorescence were taken at the same wavelengths prior to staining. Before drug administration, after the first medium exchange, 24 h after drug administration, and after fluorescence imaging cell confluence was assessed using the Tecan Spark Cyto, to account for changes in cell confluence due to drug administration and cell handling. Pyknosis was defined via a cutoff range describing the significant shift of Hoechst33342-positive signal per nuclear area in bexarotene vs. vehicle treated cells. The mean intensity/area values for each well were further normalized to the respective negative control, hence values > 100% in response to drug treatment indicate an increment of pyknosis. Metabolic activity, changes in cell confluence and necrotic cell numbers (percent of the total cell count per well) were normalized to the negative control of each biological replicate.

RE motif scanning. Genomic promoter regions were defined as 2000 bp upstream and 200 bp downstream of transcription start sites (TSS) according to community criteria[8–11], extracted from the rat genome (UCSC rn6) using RefSeq transcript annotations, restricted to standard chromosomes (1-20, X, Y, M), trimmed to chromosome boundaries, and retrieved as DNA sequences. Position weight matrices (PWMs) for the human NBRE and DR5 were taken from

JASPAR[12] (release 2024), namely MA0160.2, MA0160.3, MA1112.1, MA1112.2, and MA1112.3 to cover NBRE, and MA1147.1 and MA1147.2 to cover DR5. Extracted promoter sequences of the selected genes of interest were scanned for transcription factor binding sites using each JASPAR PWM individually. Background base frequencies were estimated from the target set and used to generate 1000 random sequences as an empirical null model. For each extracted promoter and each NR4A-related PWM, forward and reverse complement motifs were scored across all possible positions on both genomic strands, with a threshold defined as the 99th percentile of null sequence scores to filter for strong matches. Detected hits were collected and aggregated across binned promoter regions (width = 5 bp) yielding genomic region and strand specific hit counts for which empirical hit probabilities (p_{emp}) relative to the null model were calculated. Hits per promotor-PWM-pair with a p_{emp} >= 0.8 were summed up across both genomic strands and the minimum p_{emp} for each pair was selected. To arrive at a more general conclusion, JASPAR PWMs were then grouped into their regulatory element classes (NBRE, DR5) and average hit counts and average p_{emp} were calculated and visualized. Data extraction, preparation and analysis was conducted using R 4.3.3 using the packages GenomicFeatures, GenomicRanges, IRanges, GenomeInfoDb, Biostrings, BSgenome.Rnorvegicus.UCSC.rn6, TxDb.Rnorvegicus.UCSC.rn6.refGene, AnnotationDbi, org.Rn.eg.db, and TFMPvalue.

NMR spectra of compounds 2–5

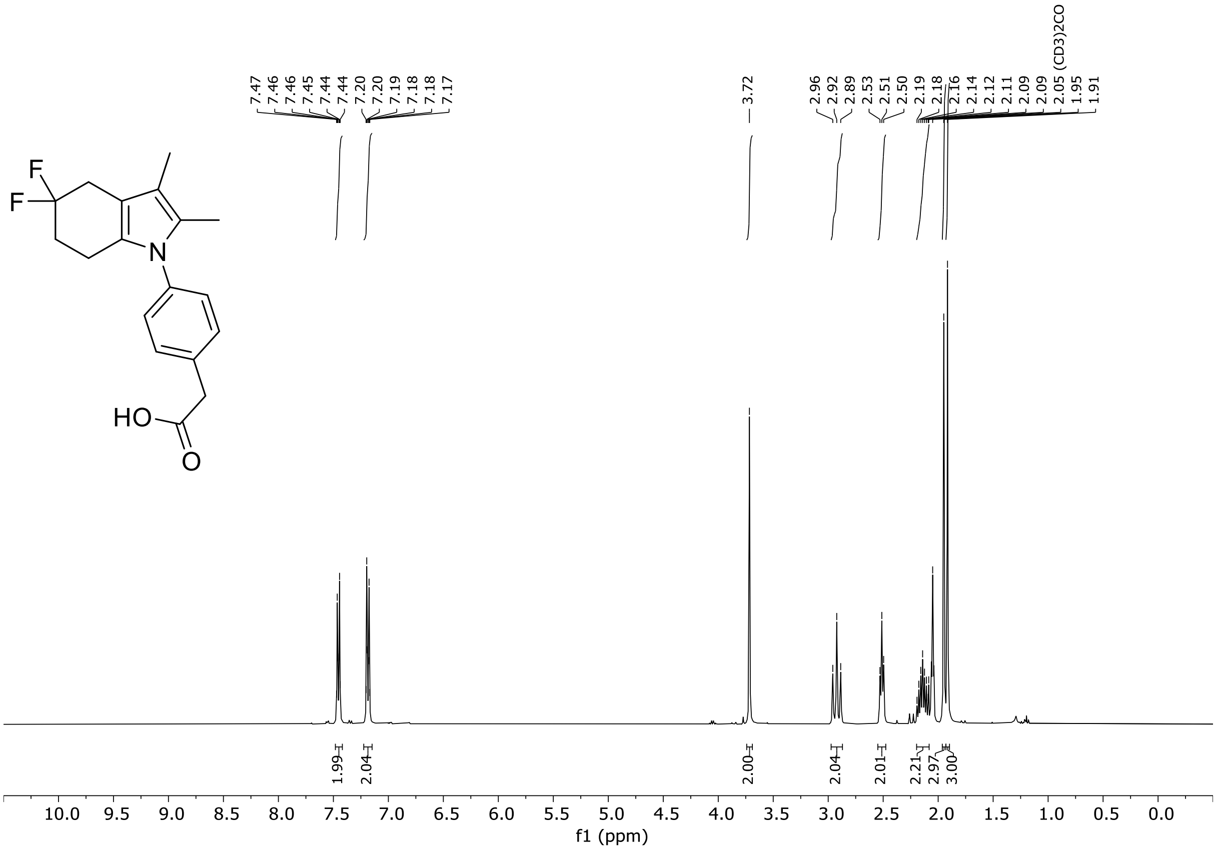

^{1}H NMR (400 MHz, acetone-d_6) of compound **2**.

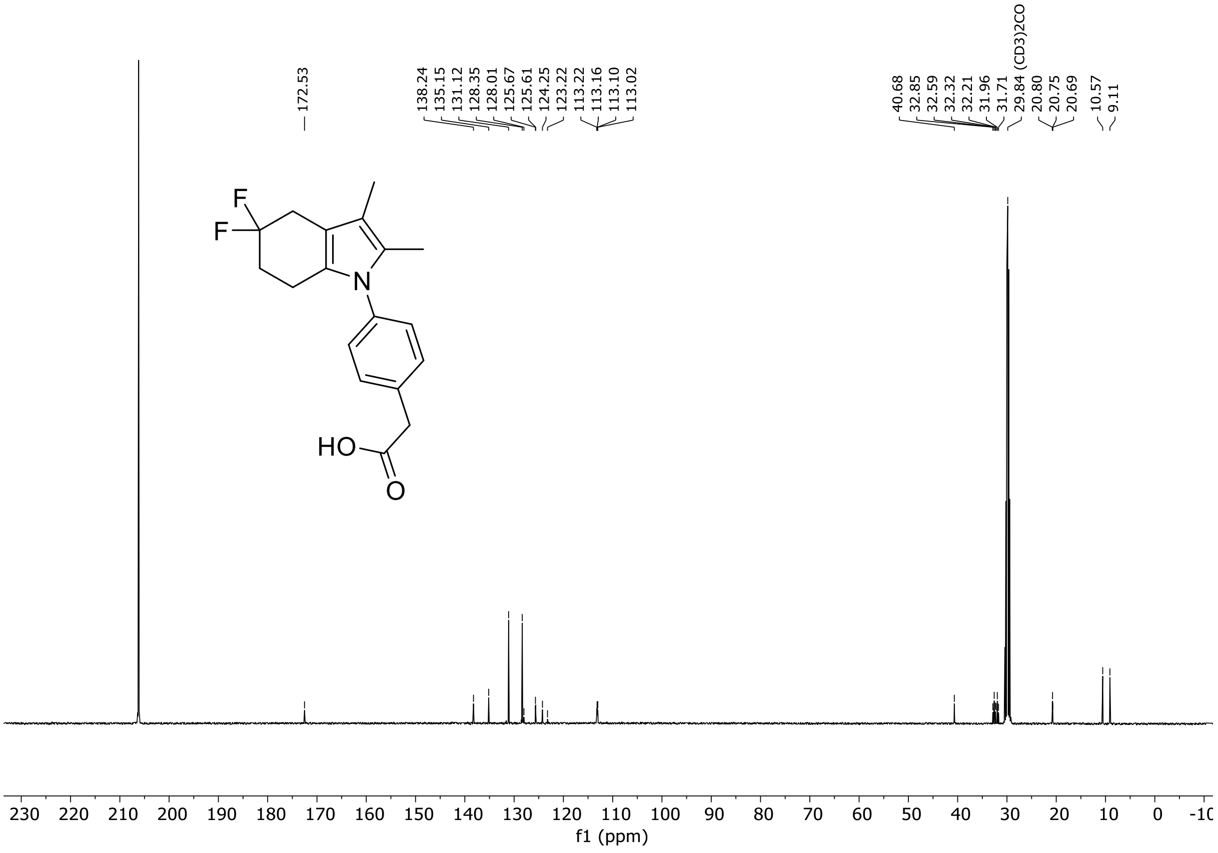

^{13}C{^{1}H} NMR (101 MHz, acetone-d_6) of compound **2**.

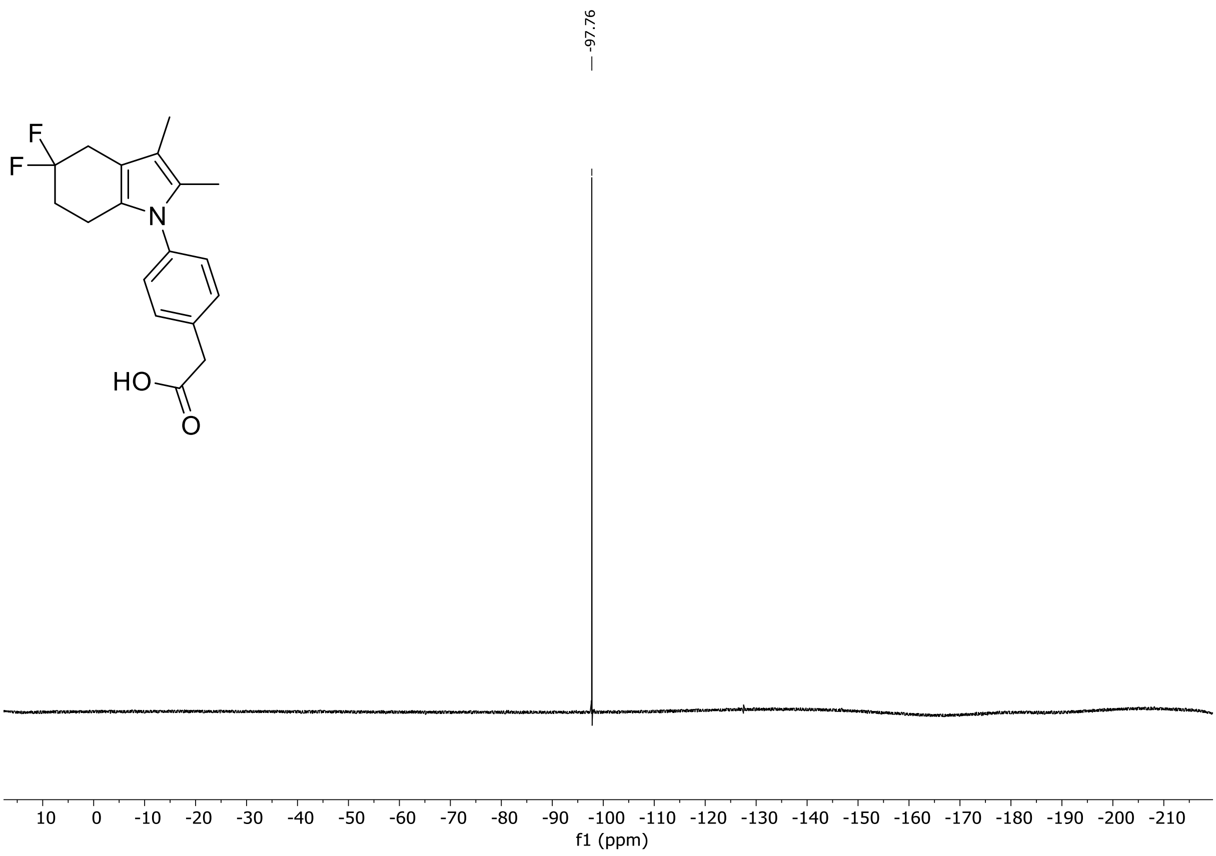

$^{19}F\{^{1}H\}$ NMR (376 MHz, acetone-d_6) of compound **2**.

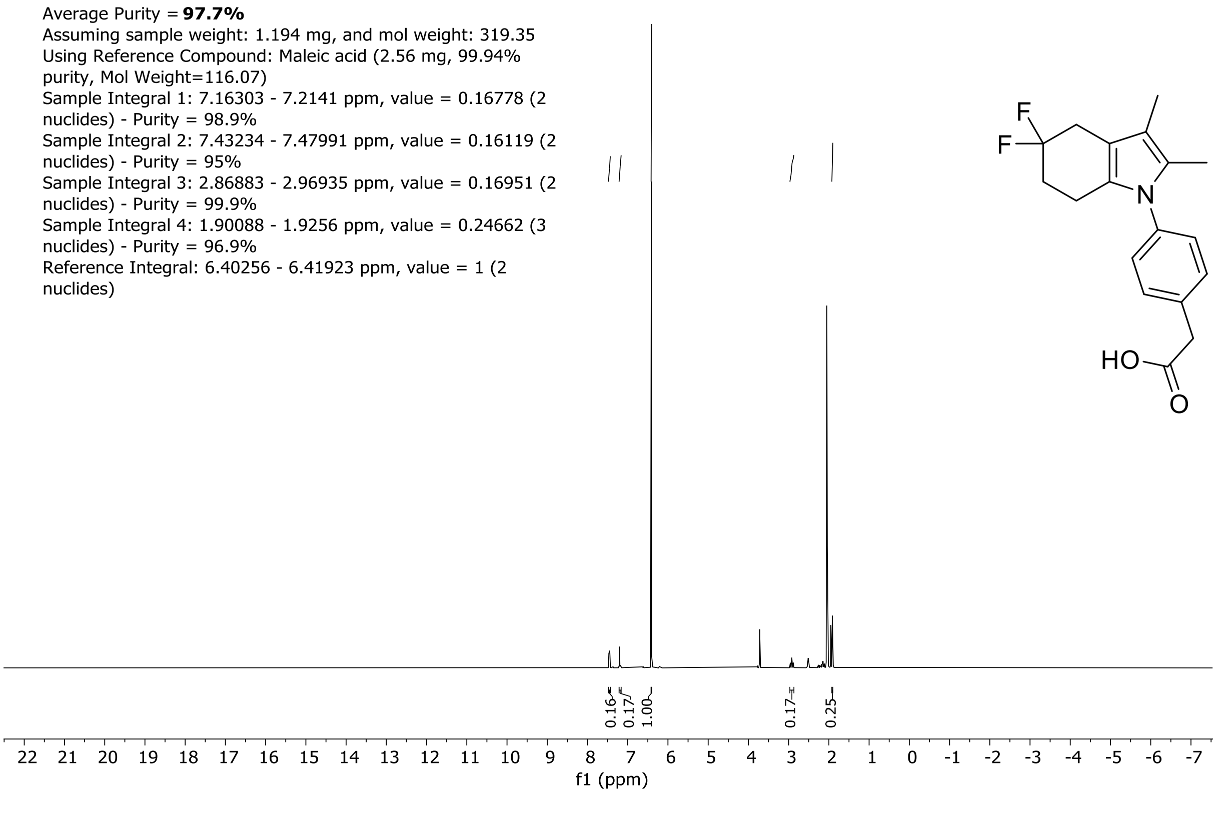

^{1}H qNMR (400 MHz, acetone-d_6) of compound **2**.

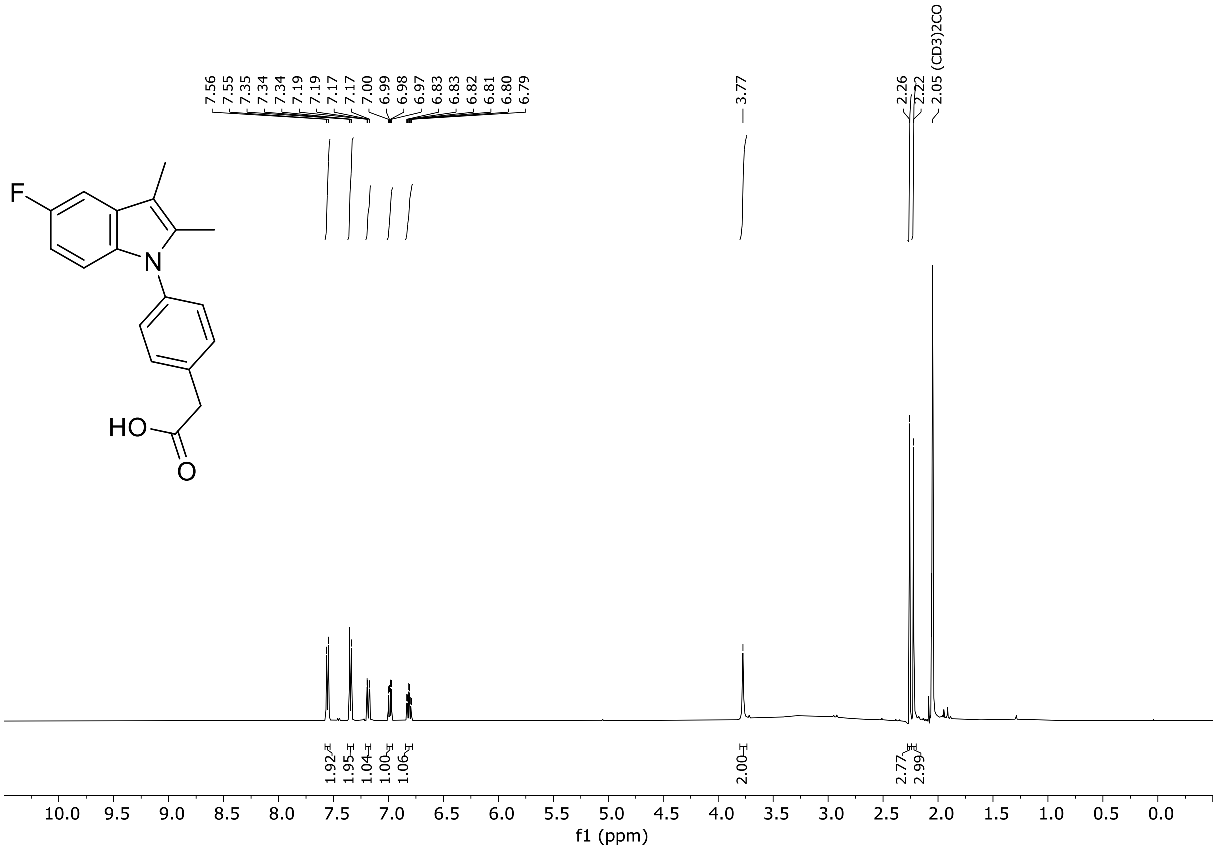

^1H NMR (500 MHz, acetone-d_6) of compound **3**.

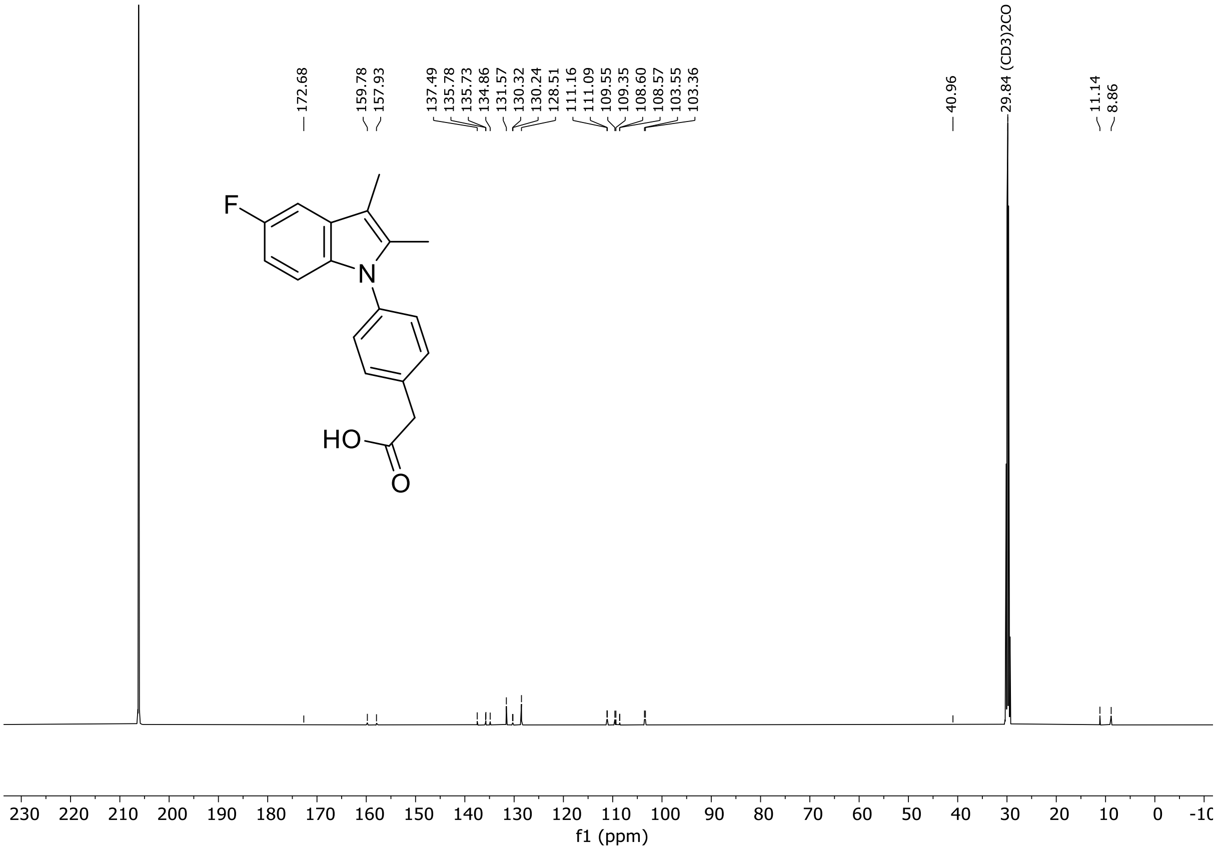

$^{13}C\{^1H\}$ NMR (126 MHz, acetone-d_6) of compound **3**.

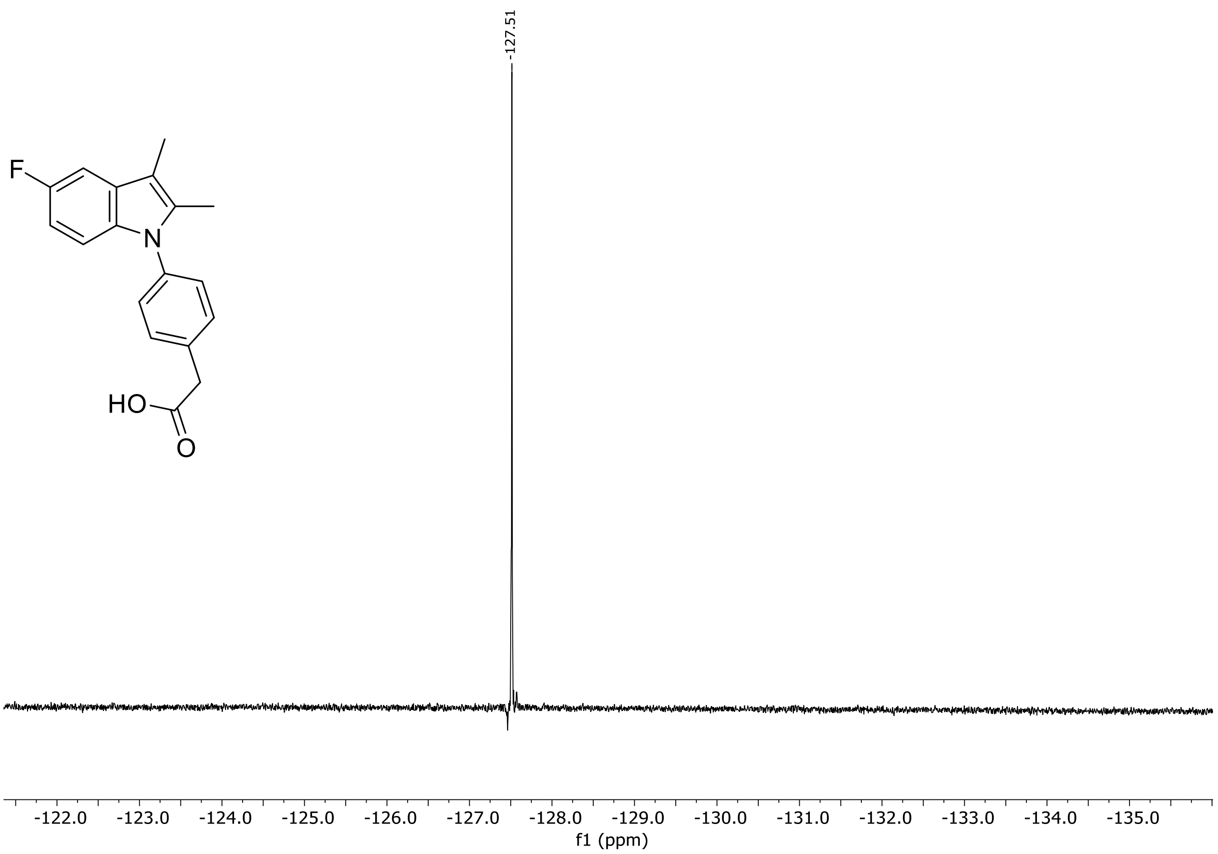

$^{19}F\{^{1}H\}$ NMR (376 MHz, acetone-d_6) of compound **3**.

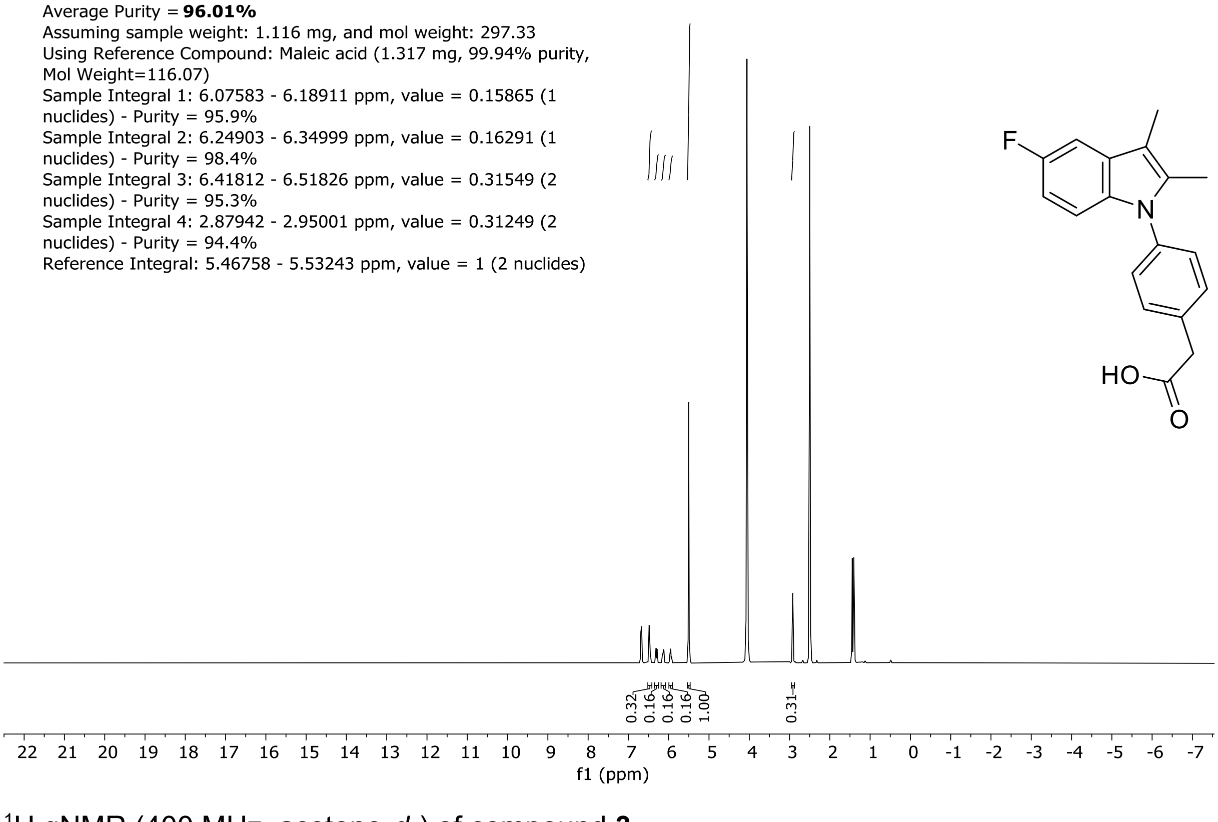

^{1}H qNMR (400 MHz, acetone-d_6) of compound **3**.

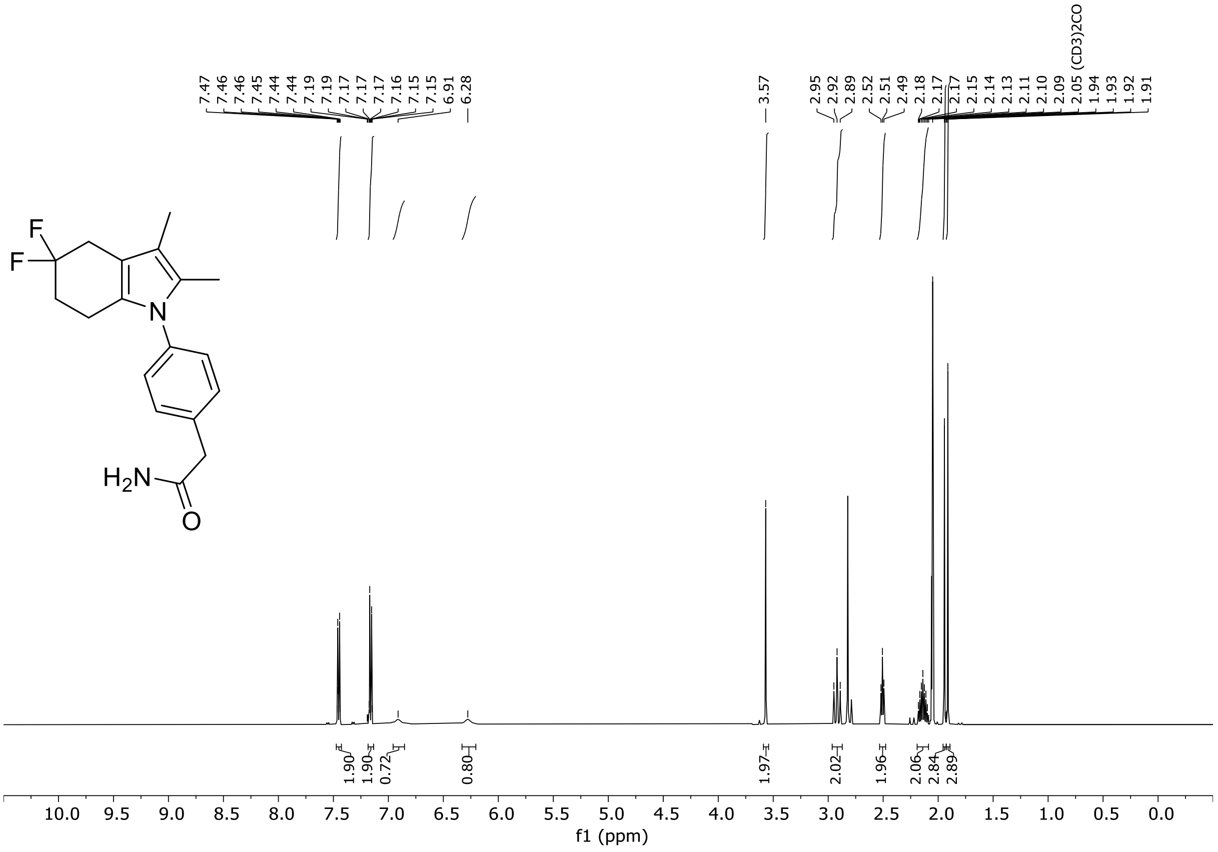

^{1}H NMR (500 MHz, acetone-d_6) of compound **4**.

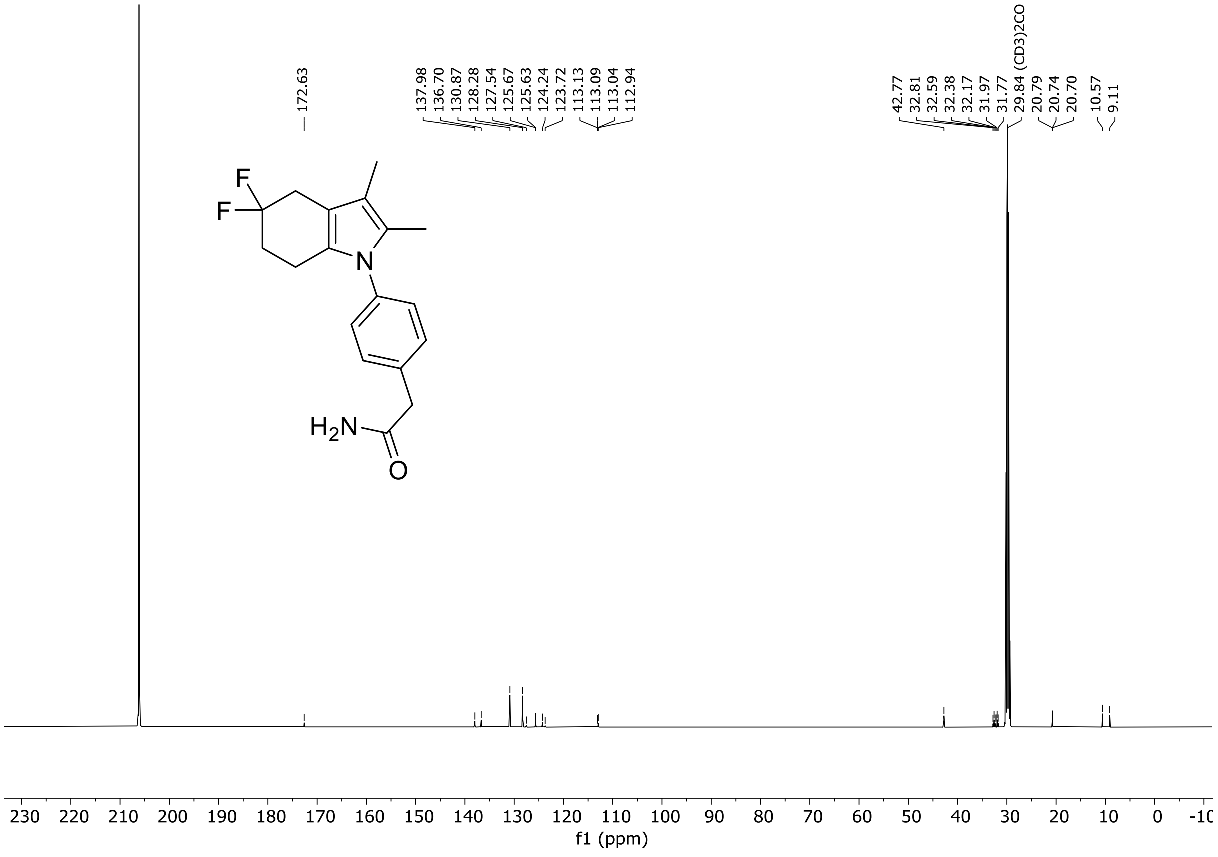

$^{13}C\{^{1}H\}$ NMR (126 MHz, acetone-d_6) of compound **4**.

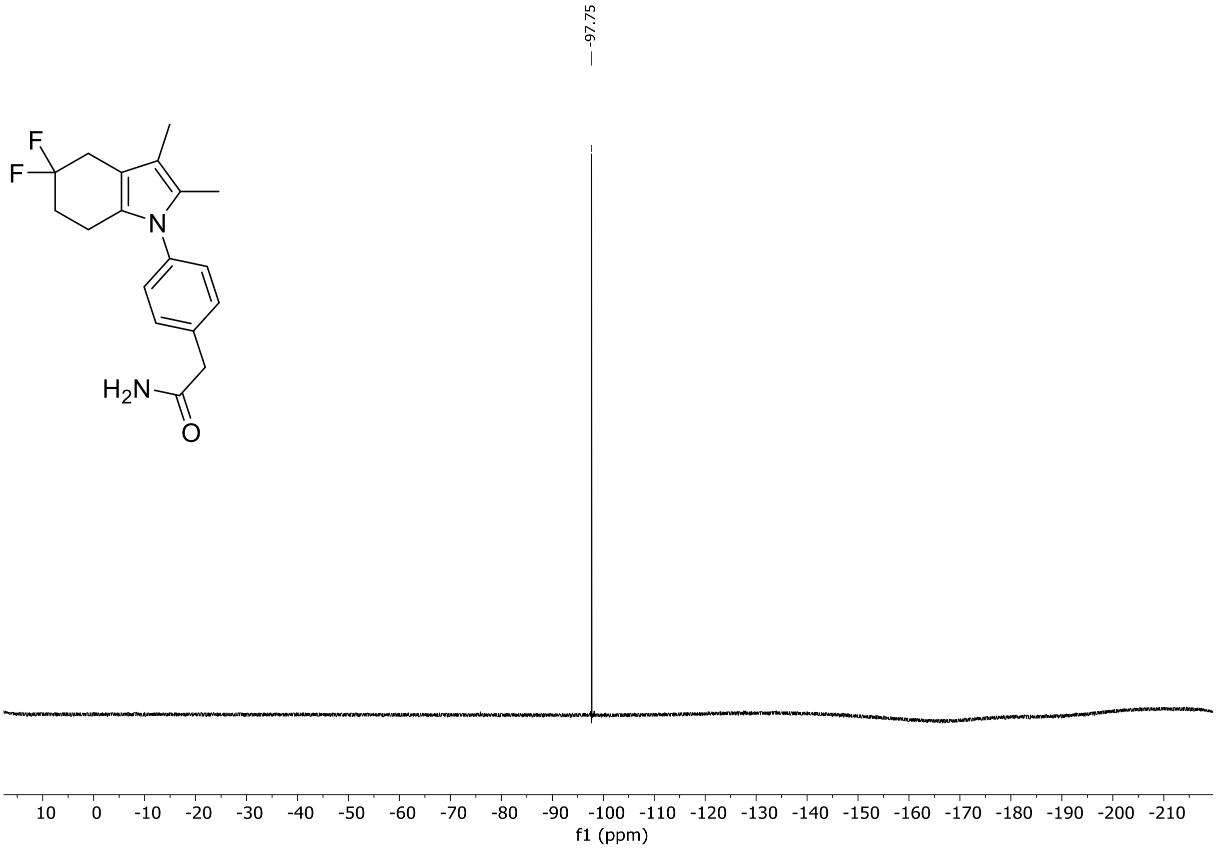

$^{19}F\{^1H\}$ NMR (376 MHz, acetone-d_6) of compound **4**.

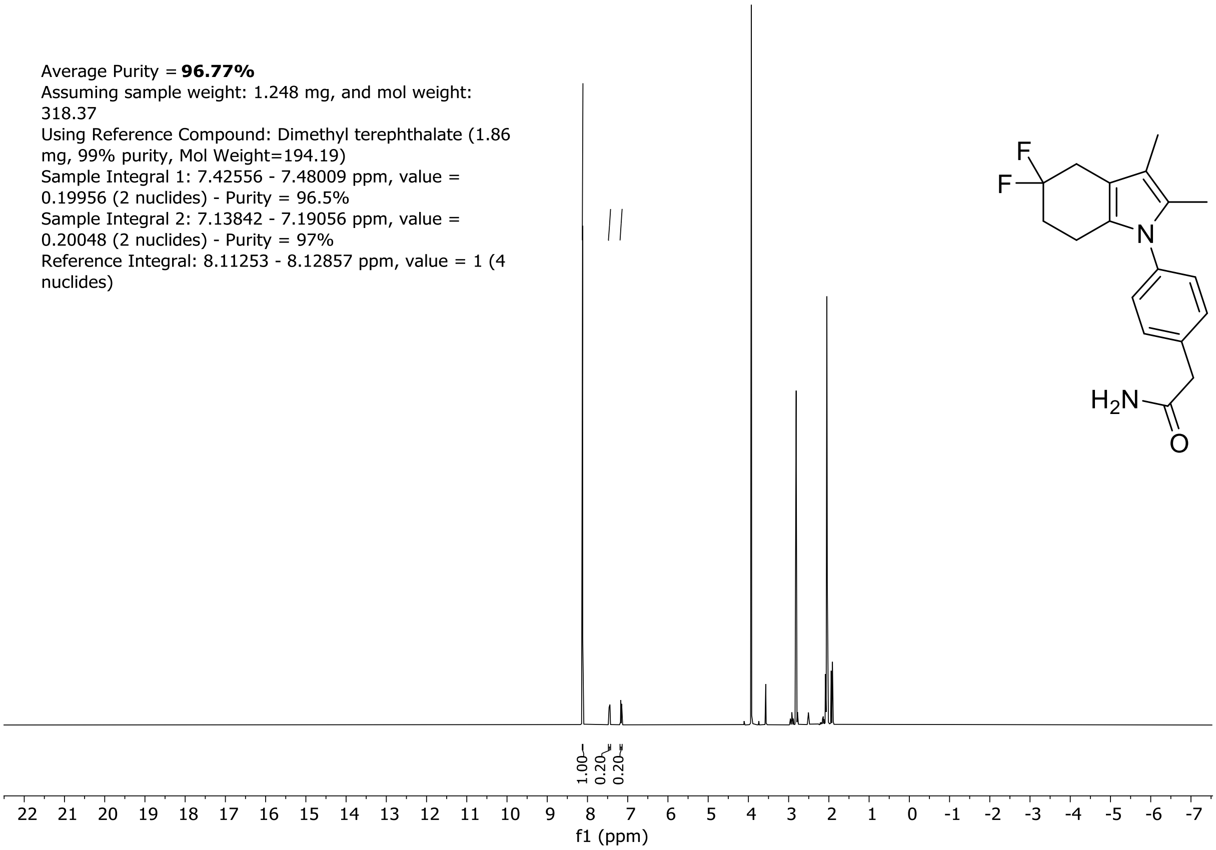

^1H qNMR (400 MHz, acetone-d_6) of compound **4**.

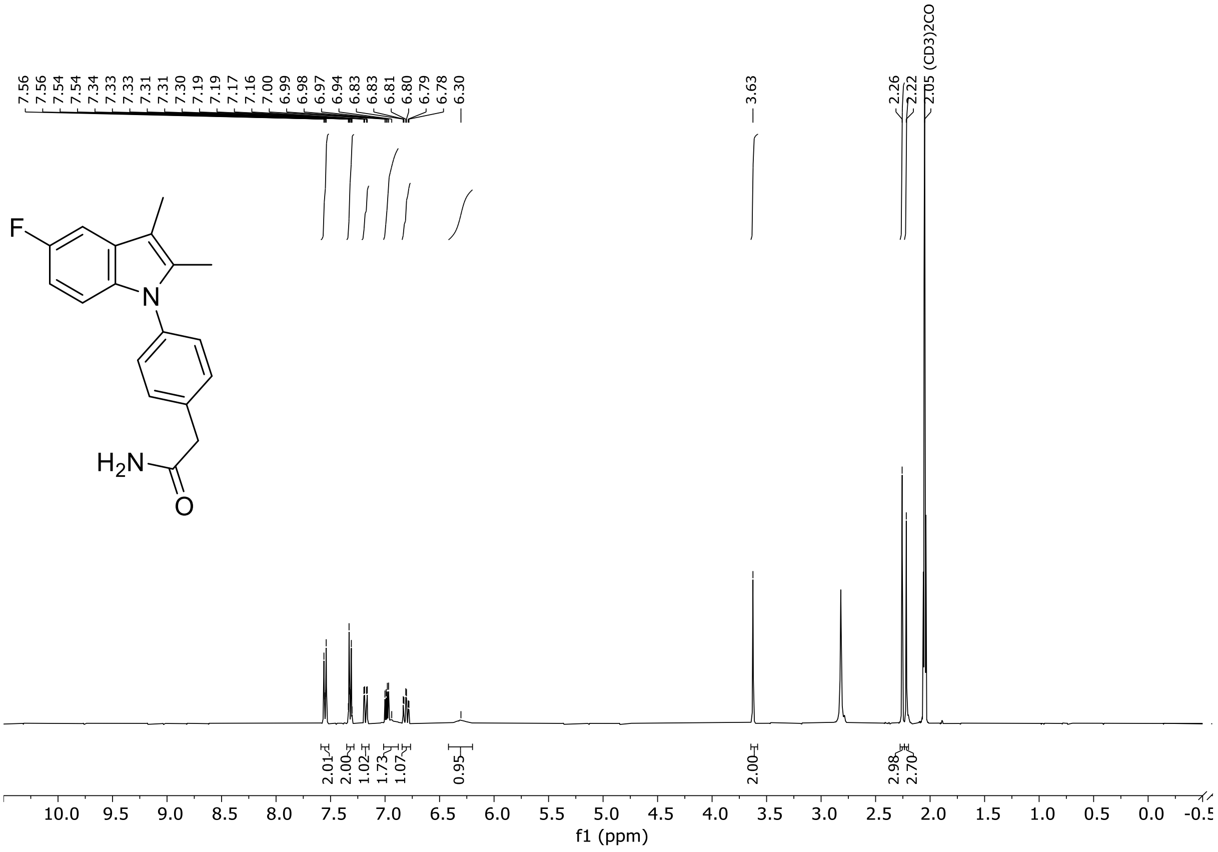

^{1}H NMR (400 MHz, acetone-d_6) of compound **5**.

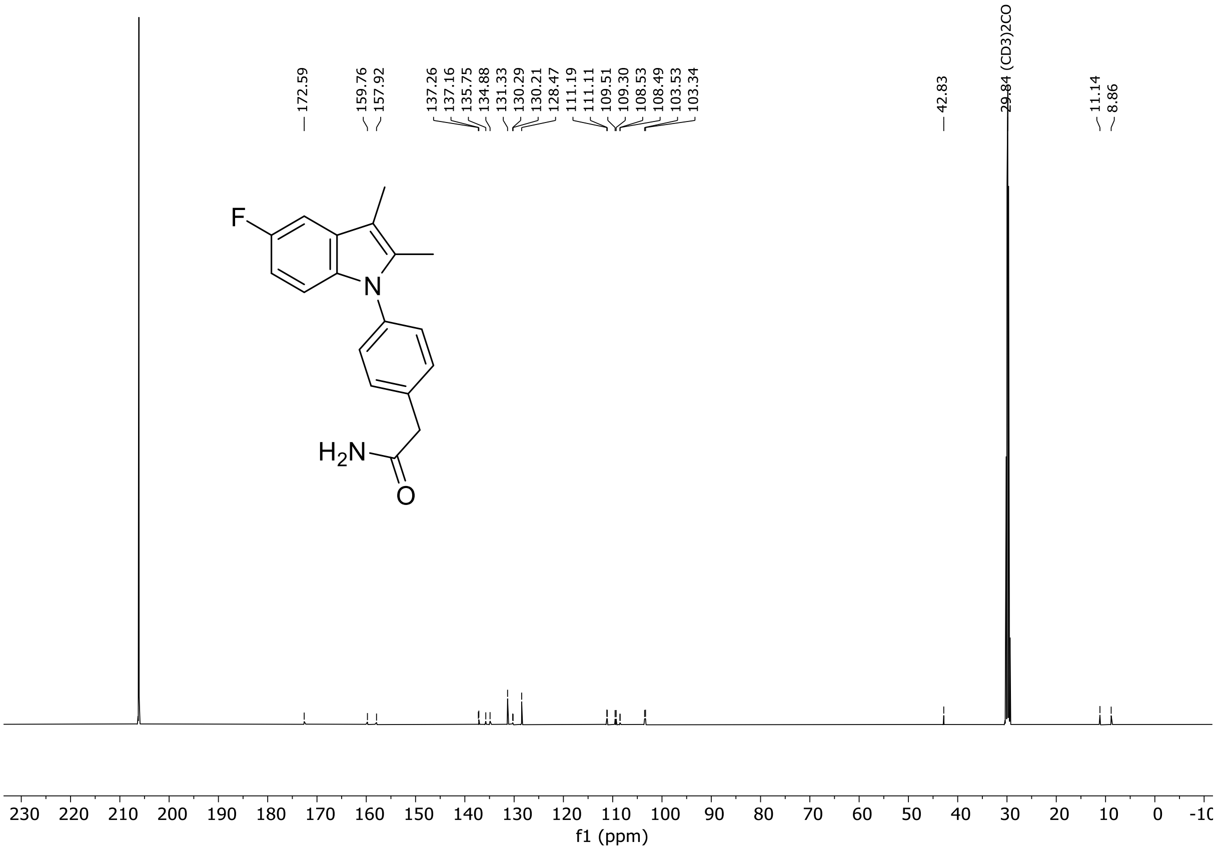

^{13}C{^{1}H} NMR (126 MHz, acetone-d_6) of compound **5**.

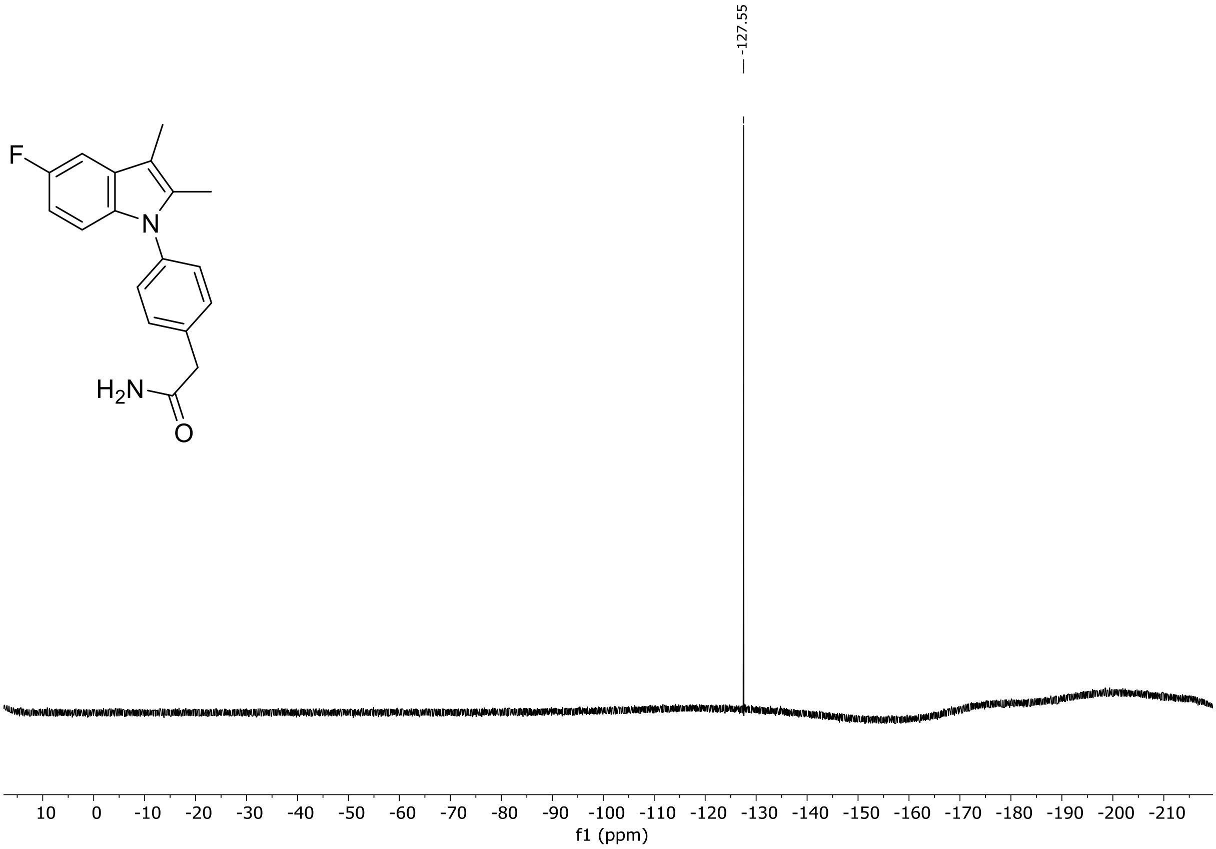

$^{19}F\{^{1}H\}$ NMR (376 MHz, acetone-d_6) of compound **5**.

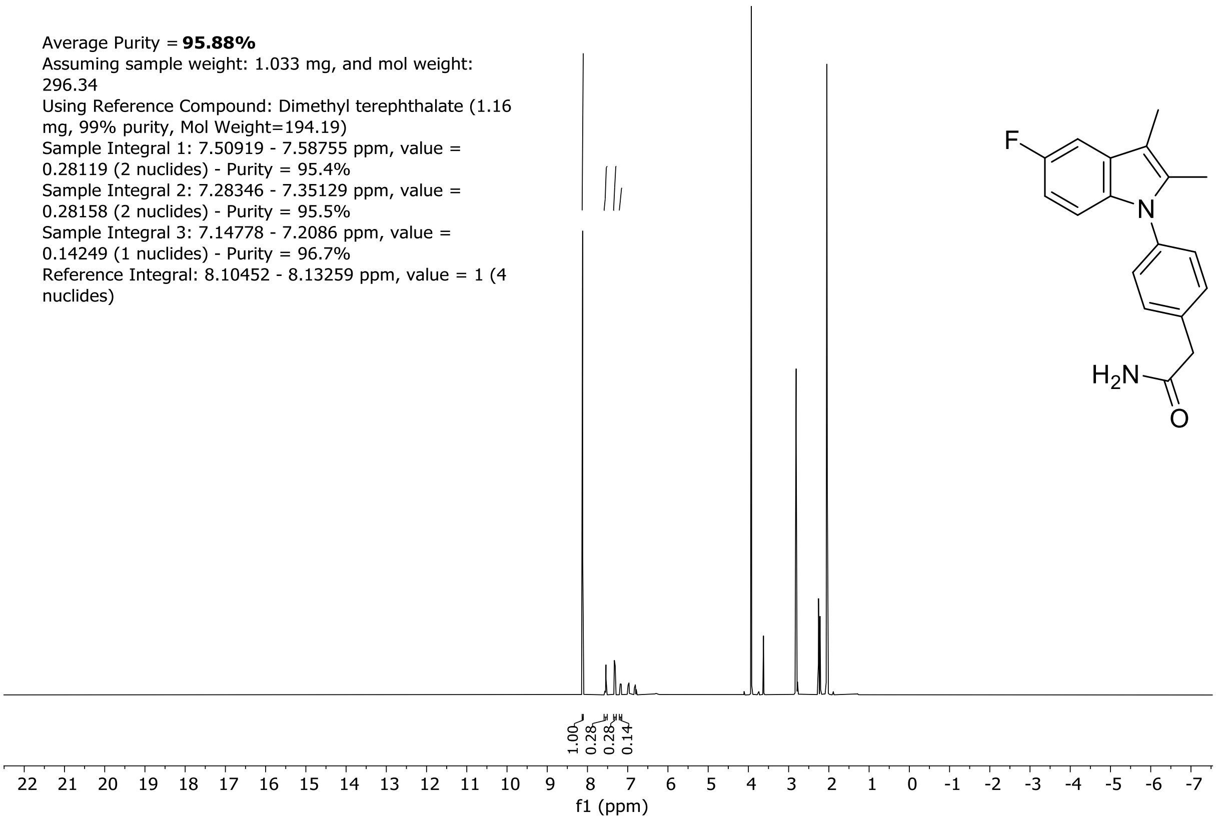

^{1}H NMR (400 MHz, acetone-d_6) of compound **5**.

Supplementary References

(1) López-García, Ú.; Vietor, J.; Marschner, J. A.; Heering, J.; Morozov, V.; Wein, T.; Merk, D. Structural and Mechanistic Profiling of Nurr1 Modulation by Vidofludimus Enables Structure-Guided Ligand Design. *Commun. Chem.* **2025**, *8*, 159.

(2) Pauli, G. F.; Chen, S. N.; Simmler, C.; Lankin, D. C.; Gödecke, T.; Jaki, B. U.; Friesen, J. B.; McAlpine, J. B.; Napolitano, J. G. Importance of Purity Evaluation and the Potential of Quantitative 1H NMR as a Purity Assay. *J. Med. Chem.* **2014**, *57* (22), 9220–9231.

(3) Barker, A. C.; Leslie, K. W.; Pervez, M.; Stanier, W. E.; Taylor, N. P. Solution-Phase Process for the Manufacture of Decapeptide. WO2003051909, 2003.

(4) Willems, S.; Kilu, W.; Ni, X.; Chaikuad, A.; Knapp, S.; Heering, J.; Merk, D. The Orphan Nuclear Receptor Nurr1 Is Responsive to Non-Steroidal Anti-Inflammatory Drugs. *Commun. Chem.* **2020**, *3*, 85.

(5) Nawa, F.; Kardanov, A.; Kasch, T.; Lewandowski, M.; Wein, T.; Höfner, G.; Marschner, J. A.; Morozov, V.; Merk, D. Development of an RXR Agonist Scaffold with Pronounced Homodimer Preference. *J. Med. Chem.* **2025**, *68*, 16172–16187.

(6) Ballarotto, M.; Willems, S.; Stiller, T.; Nawa, F.; Marschner, J. A.; Grisoni, F.; Merk, D. De Novo Design of Nurr1 Agonists via Fragment-Augmented Generative Deep Learning in Low-Data Regime. *J. Med. Chem.* **2023**, *66* (12), 8170–8177.

(7) Stiller, T.; Gege, C.; Saeb, W.; Vietor, J.; López-García, Ú.; Busch, R.; Kohlhof, H.; Vitt, D.; Merk, D. Carboxylic Acid Bioisosteres Boost Nurr1 Agonist Selectivity. *J. Med. Chem.* **2025**, *68*, 16212–16226.

(8) Håndstad, T.; Rye, M.; Močnik, R.; Drabløs, F.; Sætrom, P. Cell-Type Specificity of ChIP-Predicted Transcription Factor Binding Sites. *BMC Genomics* **2012**, *13* (1).

(9) Rye, M.; Sætrom, P.; Håndstad, T.; Drabløs, F. Clustered ChIP-Seq-Defined Transcription Factor Binding Sites and Histone Modifications Map Distinct Classes of Regulatory Elements. *BMC Biol.* **2011**, *9*.

(10) Rachid Zaim, S.; Pebworth, M. P.; McGrath, I.; Okada, L.; Weiss, M.; Reading, J.; Czartoski, J. L.; Torgerson, T. R.; McElrath, M. J.; Bumol, T. F.; Skene, P. J.; Li, X. J. MOCHA's Advanced Statistical Modeling of ScATAC-Seq Data Enables Functional Genomic Inference in Large Human Cohorts. *Nat. Commun.* **2024**, *15* (1).

(11) Bai, Y.; Deng, X.; Chen, D.; Han, S.; Lin, Z.; Li, Z.; Tong, W.; Li, J.; Wang, T.; Liu, X.; Liu, Z.; Cui, Z.; Zhang, Y. Integrative Analysis Based on ATAC-Seq and RNA-Seq Reveals a Novel Oncogene PRPF3 in Hepatocellular Carcinoma. *Clin. Epigenetics* **2024**, *16* (1), 154.

(12) Rauluseviciute, I.; Riudavets-Puig, R.; Blanc-Mathieu, R.; Castro-Mondragon, J. A.; Ferenc, K.; Kumar, V.; Lemma, R. B.; Lucas, J.; Chèneby, J.; Baranasic, D.; Khan, A.; Fornes, O.; Gundersen, S.; Johansen, M.; Hovig, E.; Lenhard, B.; Sandelin, A.; Wasserman, W. W.; Parcy, F. et al. JASPAR 2024: 20thãnniversary of the Open-Access Database of Transcription Factor Binding Profiles. *Nucleic Acids Res.* **2024**, *52* (D1), D174–D182.
